# Protein Post-Translational Modifications in the Regulation of Ferroptosis: New Opportunities and Challenges for Cancer Immunotherapy

**DOI:** 10.7150/ijbs.135689

**Published:** 2026-09-03

**Authors:** Xin Xie, Lizhou Song, Zhaoyu Li, Jiahao Hu, Zhiheng Lin, Yadong Guo, Haoling Zhang, Wangzheqi Zhang, Zui Zou

**Affiliations:** 1 School of Anesthesiology, Naval Medical University, 168 Changhai Road, Shanghai 200433, China.; 2 Faculty of Anesthesiology, Changhai Hospital, Naval Medical University, Shanghai, 200433 China.; 3 College of Basic Medical Sciences, Naval Medical University, Shanghai, 200433 China.; 4 College of Acupuncture-Moxibustion and Tuina, Gansu University of Chinese Medicine, No.35 Dingxi East Road, Lanzhou 730000, Gansu Province, China.; 5 Department of Gynecology, Longhua Hospital, Shanghai University of Traditional Chinese Medicine, Shanghai, 200032, China.; 6 Department of Urology, Shanghai Tenth People's Hospital, Tongji University. 301 Yanchang Road, Jing'an District, Shanghai 200040, China.; 7 Department of Biomedical Sciences, Pusat Kanser Tun Abdullah Ahmad Badawi, Universiti Sains Malaysia, 13200 Kepala Batas Pulau Pinang, Malaysia.

**Keywords:** ferroptosis, protein post-translational modification, cancer immunotherapy, immune escape, lipid metabolism, combined therapy

## Abstract

Ferroptosis is an emerging form of programmed cell death driven by the excessive accumulation of iron-dependent lipid peroxides, which plays a pivotal regulatory role in suppressing tumor initiation and progression. In recent years, protein post-translational modification (PTM), as a core molecular mechanism regulating protein function, stability, and subcellular localization, occupies a central position in the precise targeted regulation of ferroptosis. This review systematically summarizes and elucidates the core molecular regulatory mechanisms of ferroptosis, focusing on the dynamic regulatory pathways and effects of common protein PTM (including ubiquitination, phosphorylation, acetylation and lactylation) on key ferroptosis regulators. Meanwhile, it deeply analyzes the complex interactive regulatory network between ferroptosis and tumor immunity, and elaborates on the specific pathways by which ferroptosis remodels the tumor immune microenvironment through inducing immunogenic cell death (ICD) and releasing damage-associated molecular patterns (DAMPs). More importantly, this paper systematically discusses the potential application value of ferroptosis-related protein PTM in overcoming tumor immune escape, enhancing the antitumor activity of T cells and natural killer cells, and improving the clinical efficacy of immune checkpoint inhibitors (ICIs). Finally, it summarizes the core challenges existing in this research field and prospects the future development of novel PTM-based combination antitumor strategies targeting ferroptosis and personalized antitumor therapy. This review aims to provide new theoretical references and experimental evidence for the clinical prevention, treatment and intervention of malignant tumors.

## 1. Introduction

Cancer cells' tolerance to various cell death pathways, especially to apoptosis characterized by caspase protease activation, is the core cause of clinical tumor treatment failure and disease recurrence 1. To break through this key therapeutic bottleneck, exploring novel non-apoptotic regulated cell death modes has become a core frontier research direction in the field of current tumor targeted therapy. Against this research background, ferroptosis, a type of iron-dependent regulated cell death mediated by the abnormal accumulation of lipid peroxides, has rapidly become an emerging research focus in the field of tumor therapy since it was formally defined and named in 2012 2. Figure 1 summarizes key research milestones and the evolutionary progress of ferroptosis research since its initial discovery. Cancer cells possess core biological characteristics of abnormally active overall metabolism, excessive reactive oxygen species (ROS) accumulation, and significantly increased demand for iron ions. These characteristics determine that cancer cells are more prone to ferroptosis than normal somatic cells 3. More importantly, refractory and persistent tumor cell subsets in clinical practice that are tolerant to conventional radiotherapy and chemotherapy, such as mesenchymal-like cancer cells, dedifferentiated cancer cells, and cancer stem cells, usually show high sensitivity to ferroptosis inducers 4, 5. In summary, ferroptosis induction provides a novel strategy to overcome tumor apoptotic resistance and is expected to exert synergistic antitumor effects with conventional chemotherapy, radiotherapy and clinical immunotherapy, offering a promising therapeutic avenue for eliminating tumor lesions and improving the long-term prognosis of tumor patients.

As the core executors of life activities, proteins rely on finely tuned and dynamic regulation of protein post-translational modification (PTM) to exert their biological functions normally. PTM is a dynamically reversible biological process wherein functional chemical moieties (e.g., phosphate and acetyl groups, ubiquitin chains) are attached to specific amino acid residues via covalent bonding or enzymatic reactions following polypeptide translation on ribosomes. This process does not alter the intrinsic amino acid sequence of proteins but precisely modulates core biological behaviors, including conformational remodeling, enzymatic activity, subcellular trafficking, protein stability, and intermolecular interactions, by tuning protein physicochemical properties. A single protein can undergo multiple different types of PTM simultaneously, and various modifications can form a complex cross-regulatory network through synergistic activation or mutual antagonism, thereby realizing multi-level, dynamic, and reversible precise regulation of protein functions, which is also the core molecular mechanism for cells to respond to internal and external environmental signal stimuli 6, 7. The occurrence and development of ferroptosis are closely related to protein PTM. Various common PTM forms such as ubiquitination, acetylation, phosphorylation, lactylation, palmitoylation, and methylation can directly regulate cell sensitivity to ferroptosis at the post-translational level and participate in the molecular regulatory network of ferroptosis throughout the process 8, 9.

Cancer immunotherapy has revolutionized the clinical management of malignant tumors and has become a core antitumor modality alongside surgery, radiotherapy, and chemotherapy. However, its clinical efficacy is often significantly limited by primary or acquired immune resistance of tumors 10. One of the core strategies to overcome tumor immune resistance and improve the benefit rate of immunotherapy is to explore novel combined therapeutic targets and intervention approaches that can efficiently kill tumor cells while reversing the immunosuppressive microenvironment. Existing studies have confirmed that there is a significant synergistic anti-tumor effect between ferroptosis and cancer immunotherapy, and the two form a bidirectional regulatory interaction network: on the one hand, activated Cytotoxic T Lymphocytes (CTLs) can downregulate the expression levels of core antioxidant proteins such as Solute Carrier Family 7 Member 11 (SLC7A11) in tumor cells by secreting key cytokines such as Interferon γ (IFN-γ), block the cellular antioxidant defense pathway, and thereby sensitize tumor cells, significantly increasing their susceptibility to ferroptosis induction 11, 12; on the other hand, ferroptosis triggers the release of abundant damage-associated molecular patterns (DAMPs), which potentiate tumor immunogenicity. This process facilitates dendritic cells (DCs) maturation and antigen presentation, thereby promoting effector T cell activation and infiltration, remodeling the immunosuppressive tumor microenvironment (TME) into an immune-activated state, and rendering tumor cells vulnerable to immune attack 13.

However, in the clinical translation process of ferroptosis combined with cancer immunotherapy, it still faces severe challenges in cell-selective regulation. Although ferroptosis can efficiently induce programmed death of tumor cells, effector T cells infiltrating the TME are also highly sensitive to lipid peroxidation damage 14. Without targeted and precise regulation, non-specific broad-spectrum ferroptosis induction is likely to cause T cell exhaustion or immune dysfunction in tumor-infiltrating sites, instead reversely weakening the anti-tumor efficacy of immunotherapy, and even exacerbating the immunosuppressive state. Therefore, exploring regulatory targets and molecular mechanisms that can confer cell-type specificity to ferroptosis has become the core direction to break through the clinical translation bottleneck in this field, and protein PTM are precisely the core molecular mechanisms mediating the cell-specific regulation of ferroptosis. Existing cutting-edge studies have confirmed that tumor cells can often build a solid ferroptosis resistance barrier by regulating their abnormal protein PTM patterns, thereby escaping the precise killing of the immune system. For example, in triple-negative breast cancer, fatty acid synthase (FASN)-mediated palmitoylation of ubiquitin-specific protease 5 (USP5) strengthens the deubiquitinating effect of USP5 on glutathione peroxidase 4 (GPX4), specifically stabilizes the intracellular antioxidant system of tumor cells, and ultimately induces a hypoimmunogenic TME to suppress immune cell activation and antitumor immune responses 15. In contrast, novel targeted intervention strategies (such as the N6F11 inducer) can selectively activate tumor-specific ubiquitin ligases, precisely trigger the degradation of GPX4 in tumor cells, efficiently induce ferroptosis in tumor cells while effectively avoiding damage to normal immune cells, achieving targeted anti-tumor effects while protecting the body's immune function 16, 17. Collectively, exploring the cell-type-specific regulatory mechanisms of PTM in ferroptosis can help identify novel biomarkers for immunotherapy response and guide the development of high-specificity, low-off-target targeted drugs. This strategy enables precise tumor elimination, reverses T cell metabolic suppression and functional exhaustion, and maximizes synergistic antitumor efficacy.

This review focuses on the core regulatory mechanisms of ferroptosis for systematic elaboration, comprehensively sorts out the core molecular pathways of ferroptosis occurrence and development, and focuses on the key role of PTM of iron metabolism-related proteins in ferroptosis regulation. At the same time, it deeply explores the great clinical potential and application prospects of ferroptosis combined with cancer immunotherapy. Elucidating the intrinsic link between ferroptosis and protein PTM is essential to resolve current bottlenecks in tumor therapy. Clarifying this core molecular regulatory network can substantially optimize cancer immunotherapy and improve clinical outcomes. In contrast, broad-spectrum intervention without precise cell targeting and mechanistic regulation may cause immune cell damage and therapeutic counteraction, thereby compromising antitumor efficacy. Therefore, precisely balancing the regulatory relationship between ferroptosis-related protein PTM and cancer immunotherapy, and optimizing their synergistic intervention strategies, will be the key scientific issues and core research directions for conquering malignant tumors, improving patients' long-term prognosis, and enhancing clinical benefit in the future.

## 2. Molecular Mechanisms of Ferroptosis

### 2.1 Definition and Characteristics of Ferroptosis

Ferroptosis is an iron-dependent regulated cell death driven by excessive intracellular lipid peroxide accumulation, distinct from apoptosis, necrosis, and autophagy at morphological, biochemical, and genetic levels 2, 18. Morphologically, it primarily affects mitochondria, manifesting as mitochondrial shrinkage, increased membrane density, and reduced/lost cristae, with normal nuclear volume and no chromatin condensation (a hallmark of apoptosis) 19, 20. Cells may also exhibit necrotic morphological traits, including impaired plasma membrane integrity and variable swelling of cell bodies and intracellular organelles 20. Biochemically, the key hallmarks of ferroptosis are the accumulation of free iron and uncontrolled lipid peroxidation 2. Intracellular Fe²⁺ triggers massive ROS via the Fenton reaction and activates lipoxygenases, initiating lipid peroxidation chain reactions 18, 21, 22. Genetically, ferroptosis is modulated by sophisticated regulatory networks, among which the glutathione-glutathione peroxidase 4 (GSH-GPX4) axis serves as the central defensive pathway 23. Other protective mechanisms encompass the coenzyme Q10 (CoQ10) system 24, 25, the nuclear factor erythroid 2-related factor 2 (NRF2) antioxidant cascade 26, 27, and endosomal sorting complex required for transport III-mediated membrane repair 28. The core ferroptosis-related pathways and their regulatory crosstalk are systematically summarized in Figure 2.

### 2.2 Key Regulatory Proteins and Their Functions

Ferroptosis initiation and execution are tightly controlled by key proteins forming a network that determines cellular sensitivity, with GPX4 and SLC7A11 as the core regulatory engine 29. SLC7A11 transports extracellular cystine into the cell and mediates the efflux of intracellular glutamate 30. Internalized cystine is converted to cysteine in an NADPH-dependent reaction, and cysteine acts as an essential precursor for the synthesis of the core antioxidant GSH 31. GPX4 is the only enzyme directly reducing membrane phospholipid hydroperoxides, relying on GSH to convert toxic lipid peroxides into harmless alcohols and suppress ferroptosis 29, 32, 33. The SLC7A11-GSH-GPX4 axis thus represents the canonical anti-ferroptotic pathway, dynamically modulated by PTM like phosphorylation 34. Representative inhibitors of this axis are erastin, which targets SLC7A11, and RAS-selective lethal 3 (RSL3), which suppresses GPX4 activity 35, 36. In addition to this core axis, multiple parallel defensive systems safeguard cells against ferroptosis. Ferroptosis suppressor protein 1 (FSP1) reduces CoQ10 to the lipophilic antioxidant CoQ10H₂ on the plasma membrane 24, 25; mitochondrial dihydroorotate dehydrogenase (DHODH) sustains intracellular CoQ10H₂ abundance 37; and GTP cyclohydrolase 1 (GCH1) blocks ferroptosis through tetrahydrobiopterin (BH4)-mediated lipid remodeling 38. In ferroptosis-promoting mechanisms, Acyl-CoA synthetase long-chain family member 4 (ACSL4) and lysophosphatidylcholine acyltransferase 3 (LPCAT3) esterify polyunsaturated fatty acids (PUFAs) and transfer them to membrane phospholipids, thus supplying substrates for lipid peroxidation and increasing cell membrane susceptibility to peroxidative injury 39, 40. The lipoxygenase family, particularly arachidonate 15-lipoxygenase-1 (ALOX15), catalyzes PUFAs peroxidation and thus drives ferroptotic progression 21, 41. Importantly, the functions of these key regulatory proteins are tightly modulated at two distinct levels: transcriptional regulation by core transcription factors (e.g., pro-ferroptotic p53 and anti-ferroptotic NRF2) 42, 43, and PTM (ubiquitination, phosphorylation, and acetylation) that fine-tune protein stability, enzymatic activity, and subcellular localization 34.

### 2.3 Regulatory Network of Iron Metabolic Homeostasis

Dysregulated iron homeostasis is a prerequisite for ferroptosis, with bidirectional crosstalk between the two processes. Excess intracellular Fe²⁺ catalyzes robust ROS production via the Fenton reaction, triggering lipid peroxidation and enhancing ferroptosis sensitivity 44. The labile iron pool (LIP), a pool of catalytically active iron, is tightly regulated by iron uptake, storage, utilization, and efflux to maintain homeostasis 45, 46. Iron uptake primarily relies on the transferrin (TF)/transferrin receptor 1 (TFR1) pathway 47: plasma Fe³⁺ binds TF, is internalized via TFR1, reduced to Fe²⁺ by STEAP3 in endosomes, and transported to the cytoplasm by divalent metal transporter 1 (DMT1), elevating LIP levels 48, 49. Excess cytosolic iron is sequestered in ferritin for detoxification. Under stress, nuclear receptor coactivator 4 (NCOA4)-mediated ferritinophagy degrades ferritin, releasing bound iron and increasing LIP to drive ferroptosis 50, 51. Iron is utilized for heme and Fe-S cluster synthesis, supporting mitochondrial respiration, DNA repair, and enzymatic activity 52-54. Cellular iron efflux is exclusively mediated by ferroportin (FPN), whose function is governed by the iron regulatory protein (IRP)/iron responsive element (IRE) system 55, 56. Upregulated FPN expression decreases LIP levels and suppresses ferroptosis 57.

### 2.4 Ferroptosis and Cancer

Ferroptosis plays dual roles in cancer: it acts as a tumor suppressor while enabling malignant cells to develop drug resistance and immune evasion, making it a key target in precision oncology 58. As it relates to tumor suppression, ferroptosis acts as a natural defense pathway for the body that removes preneoplastic cells and limits malignant tumor development. Many classical tumor suppressors display their antitumor biological function by inducing and activating the ferroptosis pathway. The tumor suppressor p53 directly represses SLC7A11 transcription, which blocks cystine uptake and GSH synthesis, thereby inducing ferroptosis in cancer cells. In contrast, loss-of-function p53 mutants completely abrogate this ferroptosis-inducing activity and fail to suppress tumor growth via ferroptosis 42. In a similar vein, the deubiquitinase BRCA1-associated protein 1 (BAP1) represses SLC7A11 transcription through epigenetic modulation and decreases its protein expression level, promoting ferroptosis in tumor cells with an antitumor effect 59. SLC7A11 knockout suppresses KRAS-mutant pancreatic ductal adenocarcinoma, confirming the anti-tumor effect of blocking ferroptosis escape 60, 61. Radiotherapy and immune checkpoint inhibitors (ICIs) also exert anti-cancer effects partially via ferroptosis induction 62, 63.

Conversely, cancer cells evade ferroptosis by upregulating anti-ferroptotic pathways. SLC7A11 is dysregulated in many malignant tumors, like lung cancer, triple-negative breast cancer and pancreatic cancer 64. By abundantly assimilating extracellular cystine and reducing it to cysteine, SLC7A11 robustly stimulates GSH production, and thus activates the lipid peroxide-scavenging activity of GPX4, inhibiting the key step of ferroptosis 65, 66. Moreover, compensatory pathways including FSP1/CoQ10 and GCH1/BH4 further enhance ferroptosis resistance 67, 68.

Therapeutically, ferroptosis-targeted strategies focus on inhibiting core defenses (e.g., erastin, Imidazole ketone erastin [IKE] for SLC7A11; RSL3 for GPX4) and targeting metabolic vulnerabilities (e.g., glutamine deprivation in SLC7A11-high cancers) 5, 29, 69-71. Accumulating preclinical evidence has demonstrated that the combination of ferroptosis inducers with conventional chemotherapy, radiotherapy, targeted therapy, and immunotherapy can efficiently reverse tumor drug resistance. Notably, such combinatorial regimens exert potent cytotoxic effects on apoptosis-resistant mesenchymal-like tumor cells, offering a promising therapeutic strategy for advanced and refractory malignancies 72-74.

## 3. Types of PTM of Ferroptosis-Related Proteins

Diverse PTM endow core ferroptosis regulatory proteins with versatile functional phenotypes and activity states, enabling their fine spatiotemporal regulation and maintenance of ferroptosis homeostasis. Figure 3 systematically illustrates the major PTM types of core ferroptosis regulatory proteins and their corresponding molecular mechanisms. Table 1 summarizes the regulatory landscape of PTM on ferroptosis-related proteins, providing a comprehensive reference for future mechanistic investigations.

### 3.1 Ubiquitination

Ubiquitination is one of the most conserved and critical PTM mechanisms in eukaryotic cells. In a series of subsequent cascaded enzymatic reactions mediated by E1 ubiquitin-activating enzymes, E2 ubiquitin-conjugating enzymes and E3 ubiquitin ligases, ubiquitins are covalently conjugated to specific amino acid residues of the target proteins. Its primary function is to guide target proteins for proteasomal or lysosomal degradation, thus precisely regulating a multitude of core cellular processes such as the cell cycle, signal transduction, and stress responses 75, 76. Moreover, non-canonical ubiquitination exerts non-degradative functions. Such regulatory cascades modulate not only the proteolytic turnover of target proteins but also their enzymatic activity, protein-protein interactions, and subcellular localization, thereby mediating unconventional regulation of cellular signaling pathways 77. The reverse deubiquitination process is catalyzed by deubiquitinating enzymes (DUBs). DUBs specifically recognize and cleave ubiquitin chains from target proteins, maintaining dynamic balance of the ubiquitination regulatory network. This process stabilizes target proteins, restores normal signal transduction, and corrects erroneous ubiquitin labeling 78. In ferroptosis regulatory pathways, E3 ubiquitin ligases and DUBs directly modulate the degradation rate and stability of key ferroptotic proteins including SLC7A11, GPX4, and FSP1 by altering their ubiquitination status, thereby controlling cellular ferroptosis sensitivity.

#### 3.1.1 Ubiquitination Regulation of SLC7A11

As the core subunit of the cystine/glutamate antiporter, SLC7A11 serves as a pivotal node in ferroptosis signaling, with its protein abundance and biological function tightly modulated by the ubiquitination system. Numerous studies have demonstrated the specific catalysis of K48-linked polyubiquitination and subsequent proteasome-dependent degradation of SLC7A11 by diverse E3 ubiquitin ligases, such as tripartite motif-containing protein 26 (TRIM26) and suppressor of cytokine signaling 2 (SOCS2). This process markedly blocks cellular cystine uptake and subsequent GSH synthesis, impairs intracellular lipid peroxide clearance, and ultimately enhances cellular susceptibility to ferroptosis 79, 80. In contrast, a number of DUBs like OTU deubiquitinase 1 (OTUB1) and USP18 prevent the degradation of SLC7A11 by specifically cleaving the ubiquitin chains from SLC7A11, maintaining its high levels in cells to protect them against ferroptosis and inhibit the process of ferroptotic death 81, 82. Notably, SLC7A11 ubiquitination-mediated regulation involves both post-translational and epigenetic transcriptional mechanisms, forming a multilayered regulatory network. BAP1, a histone H2A-specific deubiquitinase for example, restricts SLC7A11 transcription and mRNA expression by decreasing ubiquitination levels of histone H2A at the SLC7A11 promoter region, thereby reducing overall SLC7A11 expression at the transcriptional level and finally promoting ferroptosis 59. Together these findings show that the ubiquitination regulatory system controls SLC7A11 expression and function in a global manner through two pathways: primary control of protein degradation as well as secondary, epigenetic transcriptional inhibition — creating a refined set of ferroptosis regulatory mechanisms.

#### 3.1.2 Ubiquitination and Stability of GPX4

The ubiquitination system precisely and bidirectionally regulates GPX4 protein stability. Out of all the possible E3 ubiquitin ligase families, multiple subtypes such as tripartite motif-containing protein 46 and F-box only protein 31 can mediate GPX4 proteasomal degradation by catalyzing degradative ubiquitination. This impairs cellular lipid peroxide scavenging capacity, accelerates lipid peroxidation chain reactions, and promotes ferroptosis 83. Notably, the linkage type of ubiquitin chains directly determines the stability and functional fate of GPX4, highlighting the high complexity of ubiquitination-mediated regulation. For example, TRIM26 mediates the K63-linked polyubiquitination of GPX4 in certain cellular microenvironmental and stress contexts. This non-degradative ubiquitination does not initiate GPX4 degradation but rather stabilizes its protein structure or alters enzymatic activity, corroborating the bidirectional and diverse regulatory roles of ubiquitination in target protein functionality 84. Conversely, multiple DUBs such as OTUB1 and USP family members maintain GPX4 protein abundance and enzymatic activity either by directly stabilizing GPX4 or modulating upstream regulatory targets. Such regulatory effects constitute a critical cytoprotective mechanism that enables cells to resist oxidative stress and maintain cell viability 85-89. Based on these mechanisms, interventions targeting the GPX4 ubiquitination regulatory pathway — particularly the development of specific small-molecule drugs that induce GPX4 degradation — have emerged as a promising research strategy to trigger ferroptosis in cancer cells and overcome drug resistance in oncology 90.

#### 3.1.3 Ubiquitination Regulation of the FSP1 Pathway

The FSP1-CoQ10 axis is a recently discovered GPX4-independent antioxidant pathway that plays an essential role in thwarting ferroptosis, and its activity has also been shown to be stringently regulated by the ubiquitination system. In sorafenib-treated cell models, ERK signaling upregulates the E3 ubiquitin ligase TRIM54, which mediates FSP1 ubiquitination and subsequent proteasomal degradation. This inhibits the activation of the FSP1-CoQ10 antioxidant pathway, which results in a dramatic attenuation of cellular antioxidant defense and a striking increase in the susceptibility to ferroptosis 91. In contrast, the DUB USP7 indirectly promotes FSP1 transcription and protein levels through stabilization of transcription factor JunD, which strengthens the antioxidant activity of the FSP1-CoQ10 axis to suppress ferroptosis, forming a negative regulatory feedback loop 92.

#### 3.1.4 Ubiquitination of Iron Metabolism-Related Proteins

One of the key inducers of ferroptosis is iron overload. Ubiquitination serves as a pivotal regulator of key iron metabolism processes, including cellular iron uptake, storage, and export. It modulates ferroptosis progression by precisely governing intracellular iron homeostasis. TFR1 is the main cellular iron uptake protein. The E3 ubiquitin ligase β-transducin repeat-containing protein (βTrCP) catalyzes the ubiquitination and degradation of TFR1, decreasing iron influx from outside to inside cells as well as intracellular free iron levels, thus inhibiting ferroptosis 93. Counteracting this effect, OTUB1 upregulates TFR1 transcription and translation indirectly through stabilization of iron responsive element binding protein 2 (IREB2), thus promoting intracellular accumulation of iron and driving ferroptosis ultimately 94. In iron storage, ferritinophagy acts as the main pathway for regulating intracellular free iron release. The E3 ubiquitin ligase TRIM7 mediates ubiquitination and degradation of NCOA4, interrupting the ferritinophagy cascade, inhibiting the release of iron sequestered in ferritin, and lowering the intracellular LIP, thus exerting a ferroptosis-inhibitory effect 95. In iron efflux, FPN is the only known cellular iron exporter identified to date, whose stability is regulated by DUBs such as USP35. USP35-mediated FPN deubiquitination stabilizes FPN protein expression, promotes the efflux of excess intracellular iron, and relieves iron overload-induced cellular damage, thereby exerting a core cytoprotective effect 96. In addition, lipid metabolic enzymes, as important regulators of ferroptosis, are also subject to ubiquitin-dependent stability control, with ACSL4 and stearoyl-CoA desaturase (SCD) as representative examples. The ubiquitination and degradation of ACSL4, mediated by the E3 ubiquitin ligase neural precursor cell expressed developmentally downregulated protein 4-like (Nedd4L), inhibit the production of lipid peroxidation substrates and thereby block ferroptosis 97. In contrast, stabilization of SCD by the DUB USP7 enhances cellular resistance to ferroptosis by maintaining lipid metabolic homeostasis 98.

### 3.2 Phosphorylation Modification

Phosphorylation is the most common and tightly regulated type of PTM in eukaryotes. Mechanistically, protein kinases catalyze adenosine triphosphate (ATP) hydrolysis and covalently attach phosphate groups to specific amino acid residues of target proteins. Such modifications predominantly occur on serine, threonine, and tyrosine residues. This covalent modification introduces negatively charged phosphate groups into target proteins, triggering spatial conformational remodeling. Such changes directly regulate enzymatic activity, modulate subcellular localization, and mediate the assembly or disassembly of protein-protein interactions, enabling rapid and precise control of cellular signaling pathways 99. The reversible counterprocess, dephosphorylation, is catalyzed by protein phosphatases that remove phosphate groups from target proteins, restoring their native conformation and basal function or switching them to alternative functional phenotypes. Phosphorylation and dephosphorylation together form a dynamic, reversible regulatory network 99.

#### 3.2.1 Bidirectional Regulation of the SLC7A11-GPX4 Axis

Phosphorylation exerts a bidirectional regulatory effect on the SLC7A11-GSH-GPX4 canonical anti-ferroptotic axis, with both anti-ferroptotic and pro-ferroptotic activities to finely modulate the threshold of ferroptosis at the whole cell level. On one hand, phosphorylation increases cellular tolerance to ferroptosis through the stabilization of core antioxidant proteins and inhibition of degradation process. Accumulating evidence reveals that activated protein kinase B (AKT/PKB) specifically phosphorylates creatine kinase B (CKB) in hepatocellular carcinoma cells. After its modification, CKB separates from its canonical metabolic enzymatic role and adopts non-canonical protein kinase activity to directly phosphorylate serine residues on GPX4, including Ser104 100. This PTM event utilizes the binding of heat shock cognate 71 kDa protein (HSC70) to GPX4 as a physical blockade to suppress its ubiquitylation and degradation, leading to stable GPX4 protein expression and enzymatic activity, which in turn promotes tumor cells with a malignantly proliferative phenotype resistant to ferroptosis 100. However, phosphorylation can also directly inactivate cystine/glutamate antiporter system (System xc⁻), leading to the failure of the antioxidant system which promotes ferroptosis. When cells are under energy stress or treated with the ferroptosis inducer erastin, activated AMP-activated protein kinase (AMPK) phosphorylates multiple serine residues on Beclin 1 (BECN1). Oligomerized BECN1 specifically binds to SLC7A11 and forms a protein complex that directly suppresses the transport activity of SLC7A11. This leads to impaired cellular cystine uptake, GSH depletion and blockade of the lipid peroxide clearance system, which finally promotes ferroptosis progression 101.

#### 3.2.2 Positive Feedback Mechanism of ACSL4 Phosphorylation

ACSL4 is a major effector regulating lipid metabolism and driving ferroptosis. Its phosphorylation amplifies lipid peroxidation signals via a positive feedback loop and prominently drives ferroptosis 102. Mechanistic studies identified protein kinase C beta II (PKCβII) as directly targeting the Thr328 residue of ACSL4 and mediating its phosphorylation. This modification significantly enhances the catalytic activity of ACSL4 and promotes PUFA-CoA assembly, providing abundant substrates for lipid peroxidation cascades and further accelerating ferroptosis 102. Importantly, the PKCβII activation relies on early-on lipid peroxides produced intracellularly. The rapid upregulation of lipid peroxidation signals triggers the kinase to enhance pro-ferroptotic effect by phosphorylating ACSL4, which generates a positive feedback loop consisting of lipid peroxidation-PKCβII activation-ACSL4 phosphorylation-enhanced lipid peroxidation to sustainably amplify ferroptotic signals 102. It is worth emphasizing that phosphorylation regulation exhibits high substrate specificity: modifications of distinct substrates by the same kinase family can elicit diametrically opposite biological effects. For example, PKC-mediated phosphorylation of heat shock protein beta 1 (HSPB1) at Ser15 inhibits ferroptosis by stabilizing the cytoskeleton and reducing cellular iron uptake, fully illustrating the complexity and diversity of phosphorylation in ferroptosis regulation 103.

#### 3.2.3 Phosphorylation Regulation of Iron Metabolism-Related Proteins

The phosphorylation of the core iron metabolism proteins is a critical molecular mechanism that drives intracellular iron homeostasis dysregulation and then modulates cellular sensitivity to ferroptosis by controlling multiple important processes. Previous studies have verified that the kinase encoded by the Ataxia Telangiectasia Mutated (ATM) gene directly phosphorylates NCOA4 at the Ser550 residue. This modification considerably increases the specific affinity between NCOA4 and ferritin, effectively activates indirect signal - the ferritinophagy program, which hastens the release of Fe bound to ferritin to boost LIP levels in the cytoplasm and promote ferroptosis through iron deposition 104. Moreover, the phosphorylation state of ferritin itself alters its binding affinity for iron ions and the stability of proteins directly, thereby finely tuning this dynamic equilibrium in cell for iron storage and release and impacting indirectly on ferroptosis threshold. Moreover, the phosphorylation status of Yes-associated protein/Transcriptional co-activator with PDZ-binding motif (YAP/TAZ), which serve as core effectors of Hippo signaling pathway, is tightly controlled by cellular density and determines transcriptional activity through downstream target genes. As part of these, the Transferrin Receptor (TFRC) gene is a crucial regulatory target. At the core, YAP/TAZ regulates iron homeostasis and ferroptosis susceptibility via TFRC transcription modulation to change cellular iron uptake ability 105, 106.

#### 3.2.4 Context Dependency of Phosphorylation Modification

Phosphorylation can have either promoting or suppressive effects on ferroptosis, which are highly context-specific rather than a rigid biological outcome. The overall pro-ferroptotic or anti-ferroptotic effect is defined by the cellular stress microenvironment, its metabolic state and the identity of the substrate protein involved, highlighting the high flexibility and complexity of this regulatory network. A representative example exemplifying the significant bidirectional regulation of ferroptosis by PKC kinase family: it can enhance lipid peroxidation and promote ferroptosis via phosphorylating ACSL4, or inhibit the occurrence of ferroptosis by phosphorylating HSPB1 to modularize cellular structure and reduce iron uptake 102, 103. Similarly, the regulatory effects of AMPK are also context-specific. Under conventional energy stress or erastin induction, AMPK promotes ferroptosis by phosphorylating BECN1 to suppress System xc⁻ function and trigger GSH depletion. In contrast, under the specific metabolic stress of glucose starvation, AMPK inhibits ferroptosis by phosphorylating acetyl-CoA carboxylase to block the polyunsaturated fatty acid synthesis pathway and reduce the supply of lipid peroxidation substrates 101, 107. The above mechanisms fully demonstrate that cells can flexibly adopt appropriate ferroptosis regulatory modes through dynamic phosphorylation-dephosphorylation control in response to extracellular stress signals and intrinsic metabolic status, thus maintaining the homeostatic balance between cell survival and death. These findings indicate that cells dynamically adjust ferroptosis sensitivity via reversible phosphorylation modification to adapt to external stress and internal metabolic status, balancing cell survival and death. Future studies should further explore context-specific phosphorylation regulatory pathways of ferroptosis and the therapeutic potential of key kinases and phosphatases, providing theoretical basis for precision tumor therapy and ferroptosis-targeted drug resistance reversal strategies.

### 3.3 Acetylation Modification

Acetylation is one of the most conserved and important PTM in eukaryotes. Its main mechanism is acetylation of lysine residues in target proteins, catalyzed by acetyltransferases. This alteration makes the spatial conformation, stability, catalytic activity, subcellular distribution and protein-protein interactions of target proteins tightly regulated, broadly participating in core biological processes including metabolic adaptation of cells to diverse conditions, stress response regulation and determination of cell fate 108-110. Collectively, mounting evidence has demonstrated that acetylation acts as an essential core regulator driving the complex ferroptosis network. In recent years, acetylation has also been identified as another critical PTM pathway modulating ferroptosis, following ubiquitination and phosphorylation 111. The acetylation levels of multiple core effector proteins in the ferroptosis pathway are precisely and bidirectionally controlled by acetyltransferases and deacetylases. By modulating key pathways including cellular antioxidant defense, lipid metabolic remodeling, iron homeostasis maintenance, and death signal transduction, acetylation ultimately determines cellular sensitivity to ferroptosis 112-114.

#### 3.3.1 Acetylation Regulation of the Mitochondrial Antioxidant Defense System

Mitochondria serve as a central organelle governing ferroptosis initiation and execution; intramitochondrial redox homeostasis directly drives ferroptotic progression, and the functional activity of mitochondrial antioxidant enzymes is also subject to direct acetylation targeting. Prior studies show that the expression level of the mitochondrial deacetylase sirtuin 3 (SIRT3), which is primarily localized in mitochondria and plays an important role in regulating GPX4 acetylation, is significantly downregulated in cellular models with acute kidney injury-induced cadmium exposure or cisplatin treatment. Such alterations significantly undermine the stability of GPX4 protein as well as its enzymatic activity for scavenging lipid peroxide, leading to disturbance of mitochondrial antioxidant defence system and consequently initiating ferroptosis in renal tubular epithelial cells, thereby aggravating renal tissue injury 115. In contrast, activation of SIRT3 by agents like honokiol or supplementing its key cofactor nicotinamide mononucleotide can effectively reverse aberrant GPX4 acetylation to restore protein stability and enzymatic activity to inhibit ferroptosis in neurons under perioperative neurocognitive disorders and cardiomyocytes under ischemia-reperfusion injury, demonstrating cytoprotective effects 115, 116. This mechanism uncovers the key function of SIRT3-GPX4 acetylation axis in mitochondrial redox homeostasis and ferroptosis resistance.

#### 3.3.2 Dual Role of p53 Protein Acetylation in Ferroptosis

As a master transcription factor at the orientation of cell fate, p53, a well-known tumor suppressor, is also an important regulator of the ferroptosis pathway and its biological functions are tightly controlled by its site-specific acetylation status. The acetylation status of canonical acetylation sites on p53 (K117, K161 and K162, and K98) directly affects its transcriptional activation activity and regulatory preference for downstream target genes, mediating distinct cell fate outcomes 112. In relevant studies, the acetylation-deficient p53 mutant p53^3KR^ completely lacks its canonical pro-apoptotic activity in tumor models like non-small cell lung cancer but retains specific inhibition of transcriptional expression of SLC7A11 and blockade of cystine uptake and GSH synthesis, further inducing ferroptosis in tumor cells for continued antitumor effect 112. Interestingly, however, while p53 mutations in other acetylation sites deactivate its ability to regulate ferroptosis like K98 (e.g., p53^4KR^), mutation of additional acetylation sites of the protein completely abrogates such activity 112. Interestingly, SIRT3 deficiency has been shown to stimulate increased acetylation of p53 in a pathological cardiac model of fibrosis that promotes ferroptosis in cardiac fibroblasts and enhances the acceleration of cardiac fibrosis 117. Together these lines of evidence suggest that p53 acetylation does not serve as a one-directional ferroptotic regulator, but rather resembles an And/Or switch governing the fine-tuning of pathophysiological malleability toward either prosurvival or pro-ferroptotic outputs with asymmetric PTM patterns dictating hallmarks for different ferroptosis endpoints.

#### 3.3.3 Acetylation Regulation of Key Enzymes in Lipid Metabolism

Lipid metabolic reprogramming represents a core hallmark of ferroptosis, and the activity, stability, and function of multiple rate-limiting enzymes in lipid synthesis and metabolic pathways are precisely targeted and regulated by acetylation. Mechanistic studies in nasopharyngeal carcinoma have confirmed that lysine residue K383 of ACSL4 undergoes site-specific acetylation, which is catalyzed by the acetyltransferase histone acetyltransferase 1 (HAT1) and reversely modulated by the mitochondrial deacetylase SIRT3-mediated deacetylation. Elevated ACSL4 acetylation essentially prevents degradation via the K48 oligoubiquitin-proteasome system mediated by the E3 ubiquitin ligase F-box protein 10 (FBXO10), significantly stabilizing ACSL4 and extending its half-life 118. This acetylation-mediated protein stabilization has two-way biological effects: on one hand, it promotes synthesis of lipid peroxidation substrates to accelerate malignant expansion and progression of tumor cells; on the other hand, it greatly sensitizes cancer cells to radiotherapy-induced ferroptosis, offering a novel target for combined approach in cancer therapy. This completely demonstrates the double regulatory role of acetylation in cancer treatment 118. In addition, SLC25A1 and ATP citrate lyase, core components of the citrate-acetyl-CoA metabolic axis, synergistically maintain sufficient supply of cytosolic acetyl-CoA to promote acetylation of the ferroptosis regulator FSP1 at residue K168. This modification stabilizes FSP1 protein expression, strengthens its antioxidant function, and enhances cellular resistance to ferroptosis 119.

#### 3.3.4 N-Terminal Acetylation and the Protein Homeostasis and Degradation Pathway

On top of the classical acetylation of lysine residues, protein N-terminal acetylation — an abundant co-translational modification which is conserved in eukaryotes — also drives the recognition and degradation of target proteins via the Ac/N-degron pathway, participating in a specific regulation model for ferroptosis and mechanistically extending our understanding of how acetylation governs ferroptosis. The membrane associated ring-CH-type finger 6 (MARCHF6), which is one of the most important E3 ubiquitin ligases, has been recognized as the core recognition and mediator factor of mammalian Ac/N-degron pathway and a key molecule bridging N-terminal acetylation to protein degradation and ferroptosis regulation 120. Interestingly, MARCHF6 has a cellular role in ferroptosis with an overall dual regulation: on the one hand, through its intrinsic Ac/N domain, MARCHF6 acts as a specific molecular sensor and substrate for anti-ferroptotic effector proteins bearing Ac/N-degron signatures (including regulator of G-protein signaling 2 and Perilipin 2). Consequently, MARCHF6 escapes suppression by anti-ferroptotic signals and facilitates ferroptosis; the other part also marks pro-ferroptotic effector proteins including Selenoprotein M, p53 and ACSL4 via an Ac/N-degron-independent pathway for degradation so as to exert inhibitory effects on ferroptosis 121. The enzymatic activity of MARCHF6 is also tightly regulated by intracellular NADPH levels, allowing it to sense cellular metabolism and redox status, mediate the balance between pro-ferroptotic and anti-ferroptotic signals, and eventually decide on whether cells will undergo stress-induced death or survival 122.

#### 3.3.5 Acetylation Regulation at the Epigenetic and RNA Modification Levels

Acetylation-mediated regulation of ferroptosis is not limited to protein functional level. Acetylation also indirectly regulates the expression of ferroptosis-related genes at epigenetic and post-transcriptional RNA levels by targeting the activities of certain transcription factors, histone modification statuses, and functions of RNA-modifying enzymes, resulting in a multi-layered and integrated network. Inhibition of lysine acetyltransferase 5 (KAT5) reduces the acetylation of histone H3 at lysine 27 within the promoter region of GPX4, condenses the surrounding chromatin structure, and decreases transcriptional activity leading to downregulation of GPX4 expression as well as promoting ferroptosis in breast cancer cells 123. Lysine 375 of JMJD6 undergoes acetylation, which impairs its intrinsic demethylase function and subsequently mediates the METTL14/N6-methyladenosine (m⁶A)/SLC3A2 signaling axis to regulate downstream target genes expression and promote ferroptosis sensitivity in lung cancer cells 124. Additionally, RNA acetyltransferase N-acetyltransferase 10 (NAT10), a novel mRNA acetylation enzyme, catalyzes the N4-acetylcytidine modification of FSP1 and SLC7A11 to stabilize their mRNA transcripts (prolonging half-lives) and upregulate translational expression levels in colorectal cancer and hepatocellular carcinoma to inhibit ferroptosis and promote malignancy 113, 125.

### 3.4 Lactylation

Lactate was long regarded as a mere glycolytic waste product with limited biological functions. The discovery of protein lactylation has fundamentally updated the understanding of lactate biology, revealing that lactate acts as a critical metabolic signal that coordinates cellular metabolism, gene transcription, and protein function via PTM to regulate cell fate 126. Recent studies have demonstrated that lactylation is involved in the dual pathways of mediating histone chromatin remodeling and non-histone functional regulation, which play a significant role in both the initiation of ferroptosis and cancer cell escape from ferroptosis, thus providing an important molecular link between derailed tumor glycolysis and resistance to ferroptosis 127.

#### 3.4.1 Histone Lactylation

Histone lactylation represents the core subtype of lactylation, occurring primarily on lysine residues of histones H3 and H4, among which histone H3 lysine 18 (H3K18) and histone H4 lysine 12 (H4K12) are the best-characterized canonical modification sites. This modification precisely regulates the transcriptional activity of ferroptosis-related target genes by altering chromatin relaxation and spatial conformation, and its regulatory effects are highly dependent on cell type and pathophysiological context 128. In inflammatory and tissue-damaging diseases such as sepsis-induced lung injury and ischemia-reperfusion injury, histone lactylation mainly promotes ferroptosis and thus exacerbates tissue damage. In a model of sepsis-associated acute lung injury, high intracellular lactate levels markedly enhance transcriptional activity of the methyltransferase-like 3 (METTL3) promoter via the G protein-coupled receptor 81/histone H3 lysine 18 lactylation (H3K18la) signaling axis. This in turn mediates m⁶A methylation of the ferroptosis core driver ACSL4, stabilizing its mRNA and increasing protein expression, ultimately triggering ferroptosis in alveolar epithelial cells and exacerbating lung tissue injury 129. Meanwhile, histone H3 lysine 14 lactylation in endothelial cells directly activates the transcription of TFR1 while downregulating the iron exporter SLC40A1, dually driving intracellular iron overload and the lipid peroxidation chain reaction to accelerate ferroptosis 130.

In stark contrast, tumor cells can actively exploit histone lactylation to establish a sophisticated ferroptosis escape mechanism, enabling malignant proliferation and therapeutic resistance. In hepatocellular carcinoma cells, H3K18la modification significantly upregulates the transcription of cysteine desulfurase, maintaining the synthesis and homeostasis of intracellular iron-sulfur clusters, thereby blocking lipid peroxidation and inhibiting ferroptosis 131. In colorectal cancer stem cells, H4K12la modification catalyzed by the acetyltransferase p300 is specifically enriched in the promoter region of the Glutamate-Cysteine Ligase Catalytic Subunit (GCLC), reinforcing the GSH synthesis pathway and constructing a robust antioxidant defense system to mediate chemoresistance and ferroptosis resistance 132. Furthermore, in triple-negative breast cancer, lactate secreted by cancer-associated fibroblasts (CAFs) drives H3K18la modification in cancer cells, which in turn upregulates the expression of the Zinc Finger Protein 64 and synergistically activates the GCH1 antioxidant pathway and the expression of Ferritin Heavy Chain 1 (FTH1), achieving efficient inhibition of lipid peroxidation and stable chelation of free iron ions to completely block ferroptosis initiation 133.

#### 3.4.2 Non-Histone Lactylation

In addition to mediating indirect epigenetic regulation through histone modification, lactylation can also directly target core effector proteins of ferroptosis and directly modulate ferroptosis pathway activity by altering the spatial conformation, protein stability, degradation rate and intermolecular interactions of target proteins. This constitutes another core mechanism by which lactylation regulates ferroptosis, and such regulatory patterns exhibit marked tumor-type specificity. In terms of regulating target protein stability, lactylation displays diametrically opposite regulatory logics in different tumors, precisely mediating bidirectional modulation of cellular ferroptosis sensitivity. In lung adenocarcinoma, massive intracellular lactate accumulation induced by ferroptosis initiation promotes site-specific lactylation of Small Ubiquitin-like Modifier 2 (SUMO2) at K11. This modification significantly weakens the binding capacity between SUMO2 and the core ferroptosis driver ACSL4, thereby accelerating the ubiquitination and degradation of ACSL4 and forming a negative feedback protective loop that helps lung adenocarcinoma cells resist ferroptotic damage and maintain cell survival 134. In gastric cancer, by contrast, lactylation of Poly(rC)-binding protein 2 (PCBP2) at K115 mediated by the long non-coding RNA BASP1-AS1 specifically blocks the recognition and binding of PCBP2 to the ARIADNE RBR E3 ubiquitin protein ligase 2, inhibits PCBP2 ubiquitination and degradation, and effectively enhances its protein stability, thus exerting an anti-ferroptotic effect to support gastric cancer cell survival 135.

At the same time, non-histone lactylation also plays a key regulatory role in activating the cellular antioxidant defense axis and mediating ferroptosis resistance. In hepatocellular carcinoma, lactylation of peroxiredoxin 1 at K67 driven by zinc finger protein 207 effectively promotes the nuclear translocation of NRF2 and activates the NRF2 downstream antioxidant signaling pathway, thereby upregulating the transcription and expression of core anti-ferroptotic factors including SLC7A11 and GPX4, and strengthening the cellular capacity to clear lipid peroxides 136. Lactylation of NOP2/Sun RNA methyltransferase 2 at K508 enhances its catalytic activity as a 5-methylcytosine methyltransferase, stabilizes GCLC mRNA transcripts, prolongs their half-life, promotes GSH synthesis, and comprehensively elevates cellular antioxidant and anti-ferroptotic capacities 137. Furthermore, the lactylation status of histone deacetylase 1 (HDAC1) serves as an important molecular switch governing ferroptosis sensitivity in tumor cells. Reducing its lactylation level at K412 effectively activates the transcription of the m⁶A demethylases fat mass and obesity-associated protein and AlkB homolog 5, which in turn accelerates the degradation of FSP1 mRNA, weakens the FSP1-mediated antioxidant defense pathway, and markedly sensitizes tumor cells to ferroptosis inducers, providing a novel target for ferroptosis-targeted tumor therapy 138.

### 3.5 Other Key Protein PTM Regulating Ferroptosis

Beyond the four well-characterized PTM (ubiquitination, phosphorylation, acetylation, and lactylation), a growing number of understudied but functionally critical PTM have been confirmed to tightly modulate ferroptosis by altering the stability, activity, and subcellular localization of key regulatory proteins 139-141. These modifications, though less extensively explored, expand the regulatory landscape of ferroptosis and merit brief integration for comprehensiveness.

SUMOylation: SUMOylation (small ubiquitin-like modification) dynamically regulates ferroptotic signaling by counteracting ubiquitination-mediated degradation or modulating protein-protein interactions 142. For instance, SUMO2/3 modification of ACSL4 at K11 weakens its binding to ubiquitin ligases, which alleviates ACSL4 ubiquitination and impairs ferroptosis resistance in lung adenocarcinoma 134.

Methylation: Arginine and lysine methylation of ferroptosis regulators exert dual effects on ferroptosis 143. Protein arginine methyltransferase 5 (PRMT5)-mediated arginine methylation of GPX4 stabilizes its protein, inhibiting ferroptosis in multiple cancers 144. In contrast, lysine methylation of SLC7A11 by EZH2 represses its transcription, promoting ferroptosis 145.

Palmitoylation: S-palmitoylation, a reversible lipid modification, regulates the membrane localization and stability of ferroptosis-related proteins 146. As previously mentioned, FASN-mediated palmitoylation of USP5 enhances its deubiquitinase activity toward GPX4, driving ferroptosis resistance in triple-negative breast cancer 15. Palmitoylation of TFR1 also increases iron uptake and ferroptosis susceptibility 147.

O-GlcNAcylation: O-linked β-N-acetylglucosamine (O-GlcNAc) modification, a nutrient-sensitive PTM, modulates ferroptosis by targeting ferroptosis associated proteins 148, 149. Elevated O-GlcNAcylation of SLC7A11 stabilizes its transporter activity, boosting GSH synthesis and ferroptosis resistance 150.

Hydroxylation: Prolyl and lysine hydroxylation of ferroptosis regulators fine-tunes their function 151. For example, prolyl hydroxylase 3 (PHD3)-mediated hydroxylation of GPX4 at P159 blocks its degradation, conferring ferroptosis resistance 151.

Collectively, these understudied PTM form a complementary regulatory network with canonical modifications, jointly determining cellular ferroptosis sensitivity. Further exploration of these PTM will deepen our understanding of ferroptosis regulation and uncover novel therapeutic targets.

### 3.6 Structural Basis, Molecular Interaction and Crosstalk of PTM in Ferroptosis

Although the above sections have systematically described the independent regulatory functions of individual PTM in ferroptosis at the phenotypic level, most current studies focus on unilateral modification effects while neglecting the structural basis of PTM modification, direct molecular protein-protein interactions, and hierarchical crosstalk among distinct PTM types. In fact, cellular ferroptosis sensitivity is governed by integrated combinatorial regulatory effects arising from multiple PTM rather than isolated single modification events 152, 153.

#### 3.6.1 Structural Basis Underlying PTM-Mediated Ferroptosis Regulation

Covalent attachment of modifying moieties alters surface charge, intramolecular hydrogen-bond networks, domain flexibility and tertiary conformation of ferroptosis-regulatory proteins, which constitutes the fundamental structural basis for changes in enzyme activity, protein stability and subcellular localization.

Phosphorylation introduces negatively charged phosphate groups to remodel the electrostatic landscape of target domains 154. PKCβII-mediated phosphorylation of ACSL4 at Thr328 triggers conformational rearrangement within the catalytic pocket, enhancing substrate affinity toward polyunsaturated fatty acid-CoA and accelerating lipid peroxidation to drive ferroptosis 102. By contrast, phosphorylation of GPX4 at Ser104 creates a hydrophobic docking groove for HSC70 chaperone binding, which sterically blocks the recruitment of E3 ubiquitin ligases and protects GPX4 from proteasomal degradation, reinforcing antioxidant capacity against ferroptosis 100, 155.

Lysine-targeted acetylation, ubiquitination and lactylation compete for residues located within flexible loop regions or ligand-binding domains 156. K48-linked ubiquitination preferentially occurs on disordered loops of SLC7A11 and GPX4; such modification triggers local unfolding and exposes degron sequences for proteasomal recognition and degradation 79. Site-specific acetylation of ACSL4 at K383 stabilizes its tertiary fold by strengthening intramolecular polar interactions, preventing FBXO10-mediated ubiquitination and extending ACSL4 half-life to sensitize tumor cells toward ferroptosis under radiotherapy stress 118.

For nuclear effector proteins such as p53, acetylation at discrete lysine clusters remodels DNA-binding domain conformation and alters promoter selectivity 157, 158. Mutations abolishing acetylation at K117/K161/K162 retain p53-mediated SLC7A11 transcriptional suppression and ferroptosis induction, whereas additional loss of K98 acetylation abrogates this regulatory function, demonstrating that modification site topography dictates downstream transcriptional output 112.

#### 3.6.2 PTM-Dependent Molecular Interactions in Ferroptosis Signaling

PTM act as reversible molecular switches that remodel binding interfaces, tune binding affinity, and control dynamic assembly or disassembly of protein complexes, thereby rewiring ferroptosis signaling cascades.

HMGB1 translocation and immune function are tightly governed by acetylation status. Hyperacetylation within nuclear localization signal domains neutralizes positive charge, weakening HMGB1 association with chromatin and karyopherins to facilitate nuclear export and extracellular release 159. After secretion, HMGB1 released by ferroptotic cells predominantly adopts an acetylated, fully reduced configuration that signals preferentially through AGER to drive macrophage polarization and immunogenic cell death (ICD) activation 160. In comparison, disulfide HMGB1 passively leaked from damaged cells engages TLR4-MD2 complexes to trigger robust pro-inflammatory cytokine production 161. Such distinct functional outcomes illustrate how combinatorial PTM patterns rewire HMGB1 receptor selection and downstream signaling upon regulated cell death.

PTM-mediated partner switching also governs autophagy-ferroptosis crosstalk. Under basal conditions without phosphorylation, BECN1 interacts with canonical autophagy regulators including BCL2 162. Upon AMPK-dependent phosphorylation, BECN1 undergoes partner switching: it dissociates from BCL2 and enhances physical association with SLC7A11, forming a complex that inhibits cystine transport activity, depletes GSH and promotes ferroptosis 101. Within iron metabolism pathways, ATM-mediated phosphorylation of NCOA4 at Ser550 strengthens its binding affinity toward ferritin heavy chains, accelerating ferritinophagy, elevating LIP and facilitating ferroptotic execution 104.

#### 3.6.3 Multilayered Crosstalk Among Distinct PTM

Multiple types of PTM frequently converge on the same ferroptosis effector protein, forming sophisticated regulatory circuits classified as competitive modification, priming sequential cascades, and synergistic network coordination.

##### Competitive modification on shared lysine residues

Many lysine residues on GPX4, SLC7A11, ACSL4 and FSP1 serve as common acceptor sites for acetylation, ubiquitination, lactylation and SUMOylation, leading to mutually exclusive occupancy 163. For ACSL4, acetylation at K383 physically occludes the recognition motif for FBXO10 E3 ligase, antagonizing K48-linked ubiquitination and protein degradation 118. In lung adenocarcinoma, SUMOylation of ACSL4 at K11 weakens ubiquitin ligase docking and stabilizes ACSL4; upon ferroptosis initiation, increased lactate drives SUMO2 lactylation, dissociating SUMO2 from ACSL4 and permitting ubiquitination-mediated degradation to establish a negative feedback anti-ferroptotic loop 134.

##### Priming sequential PTM cascades

Upstream modification often functions as a priming mark to recruit modifying enzymes and enable secondary PTM events 164. Phosphorylation of BECN1 acts as a priming signal to recruit ubiquitin ligases, further reinforcing SLC7A11 complex inhibition and persistent suppression of cystine uptake 101, 165. Similarly, phosphorylation of YAP/TAZ modulates nuclear translocation and transcriptional activity; activated nuclear YAP/TAZ may indirectly alter the abundance of modification enzymes (acetyltransferases, DUBs) via transcriptional reprogramming, which consequently shapes the PTM landscape of iron metabolism proteins such as TFRC 166, 167.

##### Synergistic and antagonistic PTM networks

Coordinated modification networks jointly calibrate ferroptosis sensitivity. Histone H3K18 lactylation drives transcriptional upregulation of ACSL4 in alveolar epithelial cells during sepsis-associated acute lung injury, while non-histone phosphorylation modifications such as ACSL4 Thr328 phosphorylation augment ACSL4 catalytic activity 102, 129. These two regulatory tiers may synergistically amplify lipid peroxidation by elevating ACSL4 protein abundance and enhancing its enzymatic function. Conversely, acetylation stabilizes FSP1 at the post-translational level, and deubiquitination indirectly elevates FSP1 transcription in melanoma cells; these two regulatory arms may cooperatively maintain FSP1 protein pools, reinforcing GPX4-independent antioxidant defense and suppressing ferroptosis 92, 119.

Collectively, structural remodeling, dynamic molecular interactions and multi-layered PTM crosstalk jointly shape an integrated regulatory landscape that governs cellular ferroptosis susceptibility. At present, most investigations rely on candidate-protein approaches, while high-resolution structural data defining how multiple modifications coexist or compete on core ferroptosis proteins remain scarce. Resolving these mechanisms will not only clarify contradictory context-dependent phenotypes reported across different cell models but also guide the rational design of selective PTM-modulating small molecules to tune ferroptosis for tumor therapy.

## 4. Ferroptosis and Tumor Immunity

PTM serve as core mediators of crosstalk between ferroptosis and tumor immune responses. Table 2 systematically summarizes the key PTM-related molecular mechanisms governing ferroptosis-tumor immunity interaction and their specific effects on the tumor immune microenvironment (TIME). On the one hand, PTM-mediated ferroptosis regulatory pathways can initiate innate immune responses by inducing ICD and promoting the release of DAMPs. On the other hand, they can profoundly reshape the TIME by modulating the differentiation, activation and functional status of tumor-infiltrating immune cells, thereby participating in key pathophysiological processes including tumor immune escape and immunotherapy response, acting as a core hub linking ferroptosis and tumor immunity 128, 168.

### 4.1 Ferroptosis-Induced ICD

In recent years, the bidirectional crosstalk between ferroptosis and the TIME, especially whether ferroptosis can act as a novel form of ICD, has emerged as a frontier research hotspot and central focus in the field of tumor immunotherapy 13, 169. The defining biological feature of ICD is that dying cells release tumor-specific antigens, but also simultaneously promote the secretion of a range of immunoadjuvant signaling molecules. These events stimulate antigen-presenting cells (APCs) and induce a specific adaptive immune response, thereby mediating anti-tumor immune effects 170. Current studies have reported both supportive evidence and controversies regarding the typical ICD characteristics of ferroptosis, indicating that the underlying mechanisms require further clarification and validation.

#### 4.1.1 Evidence Supporting the Immunogenic Potential of Ferroptosis

Multiple in vitro and in vivo studies have confirmed that ferroptosis is accompanied by release and membrane exposure of classic ICD-associated DAMPs (including high mobility group box 1 [HMGB1], ATP and calreticulin [CRT]), which serve as key signals for initiating anti-tumor immunity 171-173. Prophylactic vaccination models (gold standard for ICD assessment) have confirmed that early ferroptotic tumor cells can vaccinate immunocompetent mice and confer resistance to homologous tumor challenge, providing robust in vivo support for ferroptotic immunogenicity 174, 175. Furthermore, ferroptosis can trigger secretion of unique immunogenic molecules including core decorin and mutant KRAS protein, extending the immunomodulatory pathways of ferroptosis 176, 177. The detailed regulatory mechanisms of these DAMPs are elaborated in Section 4.2.

#### 4.1.2 Controversy and Complexity of Ferroptotic Immunogenicity

Despite supportive evidence, ferroptosis as a canonical ICD form remains disputed, with net immunomodulatory activity showing clear duality and context dependence rather than unidirectional effects. Wiernicki et al. provided key counter-evidence: ferroptotic tumor cells failed to induce anti-tumor immunity in mouse models, and even suppressed the immunostimulatory effects of co-inoculated apoptotic cells, due to inhibited antigen processing and presentation in DCs after phagocytosis 178.

These contradictory findings are governed by an intrinsic "PTM code"—combinatorial PTM of DAMPs and stage-specific lipid peroxidation byproducts that dictate the functional polarity of ferroptotic signals, as systematically detailed in Section 4.2. Discrepancies are also linked to variations in experimental systems, including ferroptosis inducer type, cell line, induction duration, and ferroptosis stage (early/late), as further discussed in Section 4.2.

#### 4.1.3 Impact on TIME and Therapeutic Implications

Although the immunogenicity of ferroptosis has generated a great deal of controversy, the mutual regulation between ferroptosis and TIME has been extensively studied; crosstalk between them is a significant breakthrough in tumor immunotherapy.

1. the one hand, ferroptosis remodels the TIME by releasing DAMPs that drive innate and adaptive immune activation, collectively shifting immunosuppressive "cold tumors" toward immune-responsive "hot tumors" 179-181. On the other hand, tumor-infiltrating immune cells reciprocally modulate ferroptosis sensitivity in cancer cells. For instance, IFN-γ secreted by activated CD8⁺ T cells signals through the janus kinase (JAK)-signal transducer and activator of transcription 1 (STAT1) pathway to transcriptionally downregulate SLC7A11 and upregulate ACSL4, forming an "immune attack-ferroptosis-immune reactivation" feedback loop that amplifies anti-tumor immunity 181-183. The detailed DAMP-specific mechanisms are elaborated in Section 4.2.

However, in the complex solid TME, such ideal anti-tumor effects are often limited by PTM heterogeneity and compensatory resistance mechanisms, preventing their full realization. Ferroptosis-associated PTM events exhibit typical bidirectional regulatory characteristics: acetylation of HMGB1 promotes its extracellular release and exerts adjuvant functions, whereas 4-hydroxynonenal (4-HNE)-mediated non-enzymatic PTM protein adducts impair proteasomal function in DCs and directly induce immunosuppression 160, 184, 185. Meanwhile, prolonged ferroptotic stress activates compensatory PTM pathways, such as stabilizing SLC7A11 protein via upregulation of deubiquitinases, leading to ferroptosis resistance and immune escape in tumor cells 183.

Based on the aforementioned mechanisms, future research on ferroptosis-related tumor therapy should break through the conventional strategy of merely inducing ferroptosis and shift toward PTM-mediated precision targeted intervention: first, focus on the spatiotemporally specific regulation of PTM to precisely amplify the immunogenic signals of ferroptosis and avoid immunosuppressive effects; second, explore the PTM modification levels of core proteins as biomarkers to predict tumor ferroptosis sensitivity and immunotherapy response rates; third, develop combination therapeutic regimens of PTM editing agents and ferroptosis inducers to break immune tolerance in the TME at the molecular level and overcome drug resistance, so as to provide a novel strategy for precision combined immunotherapy against tumors.

### 4.2 Release of Ferroptosis-Associated DAMPs and Immune Responses

The immunogenic duality of ferroptosis is essentially determined by the "PTM code" of DAMPs and stage-specific lipid peroxidation products. During ferroptosis, the temporal release and spatial distribution of DAMPs collectively form a sophisticated immunoregulatory network, whose ultimate effects depend on cell death phase, microenvironmental context, and recipient immune cell subsets.

Ferroptosis-associated classical DAMPs include ATP, HMGB1, CRT, and heat shock proteins (HSPs), each with distinct release kinetics and PTM-dependent immunological functions 171. ATP is rapidly released during the initial phase of ferroptosis and functions as a "find-me" signal: binding to the P2X7 purinoceptor on the surface of APCs promotes APC recruitment and maturation, as well as secretion of pro-inflammatory IL-1β that subsequently drives anti-tumor T cell immunity 186. However, the immunostimulatory activity of ATP is tightly regulated by both enzymatic and modification-dependent mechanisms. Ferroptosis-associated oxidative stress generates oxidized ATP (oxiATP), a PTM-altered derivative that selectively inhibits the P2X7 receptor and abrogates the pro-inflammatory and T cell-activating effects of native ATP 187, 188. Extracellular ATP is also rapidly cleaved to adenosine by the ectonucleotidases CD39 and CD73 in the TME, which further suppresses T cell function via ligation of the adenosine A2A receptor 189.

Beyond ATP, the alarmin HMGB1 represents another core DAMPs whose release and functional polarity are directly controlled by acetylation and oxidative modification. HMGB1 secretion is mediated by autophagy-dependent inhibition of HDAC, which drives acetylation of HMGB1, facilitates its nuclear-cytoplasmic translocation, and ultimately promotes extracellular release 160. Acetylated HMGB1 exerts pro-immunogenic effects by binding to the receptor for AGER on myeloid cells, promoting M1-like macrophage polarization, DC maturation, and enhanced antigen cross-presentation to activate adaptive immunity 160. In contrast, ROS-mediated cysteine oxidation switches HMGB1 to an immunosuppressive phenotype, which facilitates myeloid-derived suppressor cells (MDSCs) recruitment and upregulates Programmed Death-Ligand 1 (PD-L1) expression in the TME 160, 190-192. HMGB1 can also signal through TLR4, with its net biological effect determined by both PTM status and the specific tumor context 160.

As a canonical "eat-me" signal, CRT translocates from the endoplasmic reticulum to the plasma membrane during ferroptosis, a process modulated by phosphorylation of endoplasmic reticulum stress-related proteins 193, 194. Phosphorylation of CRT itself further enhances its cell surface translocation and phagocytic signal potency: surface-exposed CRT binds to the CD91 receptor on APCs, augmenting the phagocytosis of dying tumor cells and subsequent antigen presentation, thereby potentiating downstream anti-tumor immune responses 175, 193, 195. HSPs, especially HSP90 and HSP70, play dual regulatory roles in ferroptosis. Intracellularly, as chaperone proteins, HSPs are widely implicated in maintaining the PTM homeostasis of core ferroptotic regulators: HSP70 inhibits ferroptosis by mediating SUMOylation of hypoxia-inducible factor 1α 196; HSP90 remodels mitochondrial morphology by inducing dephosphorylation of dynamin-related protein 1 (Drp1) at Ser637, or promotes ubiquitin-mediated degradation of GPX4 by recruiting ubiquitin ligases, thus precisely modulating cellular sensitivity to ferroptosis inducers 9, 197. When cells undergo ferroptosis, HSPs are released into the extracellular space and function as DAMPs. Extracellular HSP70 and HSP90 bind to pattern recognition receptors on APCs surfaces, promoting APCs maturation and antigen presentation to enhance anti-tumor immune responses 198.

In addition to proteinaceous DAMPs, lipid peroxidation byproducts—the defining biochemical executors of ferroptosis—function as non-canonical DAMPs and constitute another critical layer of the ferroptotic PTM code 199. These electrophilic compounds, including oxidized phospholipids and 4-HNE, form non-enzymatic covalent adducts with key proteins in immune cells (e.g., via the CD36 scavenger receptor), directly modulating immune cell function 200. The immunological activity of these lipid mediators is highly stage-dependent, aligning with the sequential progression of lipid peroxidation during ferroptosis. Early ferroptosis is dominated by ALOX15-catalyzed oxidation of di-polyunsaturated fatty acid (di-PUFA) phospholipids, generating immunostimulatory oxidized species such as 1-stearoyl-2-arachidonoyl-sn-glycero-3-phosphoethanolamine hydroperoxide (SAPE-OOH) that promote APC activation and T cell priming 20. Late ferroptosis, however, involves 15LOX-PEBP1 complex-mediated oxidation of mono-PUFA phospholipids, producing immunosuppressive oxidized phospholipids (e.g., oxPAPC) that drive inflammasome hyperactivation and IL-1β overproduction with tolerogenic outcomes 20, 24. 4-HNE, a major degradation product of lipid peroxides, further amplifies immunosuppression by forming covalent adducts with key antigen-presenting proteins in DCs, inhibiting phosphorylation-dependent activation of the nuclear factor kappa-light-chain-enhancer of activated B cells (NF-κB) pathway and reducing secretion of pro-inflammatory cytokines such as interleukin 12 (IL-12) 201, 202. Ferroptosis also upregulates cyclooxygenase-2 expression and promotes production of immunosuppressive lipid mediators including prostaglandin E₂ (PGE₂), which directly counteract the immunostimulatory effects of protein DAMPs and further exacerbate the immunosuppressive TME 29, 203-205.

Collectively, the combinatorial effects of DAMPs PTM status and stage-specific lipid peroxidation profiles give rise to pronounced spatiotemporal heterogeneity in ferroptotic immunogenicity. Ferroptotic cells in the early stage release a variety of immunostimulatory DAMPs such as ATP and HMGB1, triggering enhanced activation of APCs and adaptive immunity in addition to presenting features of ICD 13. However, with the progression of ferroptosis, late-stage dying cells may lose immunostimulatory power gradually and have even been reported to switch to an immunosuppressive phenotype due to the depletion of various DAMPs or accumulation of immunosuppressive lipid metabolites as well as via impaired antigen presentation by APCs after phagocytosing dead cell debris 178. Moreover, the ultimate immunological effects of DAMPs are considerably influenced by cell type, ferroptosis inducer type, TME and more^206^. Thus, ferroptosis does not have an immune activation or immune suppression feature per se; its immunological consequence is a dynamic result of the competition/superimposition/conversion of different DAMPs under certain spatiotemporal settings through PTM-mediated signaling cascades. A deep insight into this complexity is greatly important for the design of temporally controllable, cell-specific ferroptosis-targeted therapeutic strategies or immunomodulators-coadministrated therapeutic regimens.

### 4.3 Effects of Ferroptosis on Immune Cell Functions

As a critical metabolic cell death pathway, ferroptosis also modulates the function and polarization state of a variety of immune cells 207. Its immune regulatory effects are not simply stimulatory or inhibitory but exhibit prominent cell-type and context dependence. The immunomodulatory effects of ferroptosis and the underlying molecular mechanisms in different immune cell subsets are illustrated in Figure 4.

#### 4.3.1 Innate Immune Cells

##### Tumor-Associated Macrophages

Polarization of Tumor-Associated Macrophages (TAMs) is closely linked to ferroptosis susceptibility. M1-type (pro-inflammatory/anti-tumor) TAMs highly express inducible nitric oxide synthase (iNOS) and generate nitric oxide radicals (NO•). This reactive molecule effectively inhibits the lipid peroxidation chain reaction by covalently modifying or competitively inactivating the key lethal enzyme ALOX15 208. Further studies have demonstrated that iron overload-induced ROS accumulation promotes p53 acetylation, thereby driving macrophage polarization toward the M1 phenotype 209. Conversely, in certain models, JAK2 phosphorylates signal transducer and activator of transcription 3 (STAT3) to initiate hepcidin transcription, which mediates FPN degradation and sustains intracellular iron overload, ultimately triggering ferroptosis 210, 211. In contrast, M2-type (anti-inflammatory/pro-tumor) TAMs are more vulnerable to ferroptosis due to low iNOS/NO• levels 208. Accordingly, the selective elimination of M2-type TAMs or their repolarization toward the M1 phenotype using ferroptosis inducers is regarded as a potential strategy to reverse the immunosuppressive TME. However, this process is highly complex: ferroptotic TAMs have been found to release pro-angiogenic or immunosuppressive factors, and oxidized lipids derived from polymorphonuclear myeloid-derived suppressor cells (PMN-MDSCs) can induce ferroptosis in TAMs and enhance their pro-tumor functions, highlighting the critical role of intercellular communication in this process 212.

##### Neutrophils/PMN-MDSCs

PMN-MDSCs represent a key hub through which ferroptosis modulates the immune system. In the TME, PMN-MDSCs undergo ferroptosis owing to downregulated GPX4 expression driven by high levels of fatty acid transport protein 2 or hypoxic conditions 212. Specific PTM mechanisms confer ferroptosis resistance in subsets of neutrophils: for instance, in the metastatic microenvironment, itaconate produced by neutrophils in response to tumor-secreted granulocyte-macrophage colony-stimulating factor directly modifies Kelch-like ECH-associated protein 1 (KEAP1) via covalent alkylation, thereby activating NRF2-driven antioxidant defense 213. Additionally, some MDSCs stabilize the p53 protein by highly expressing N-acylsphingosine amidohydrolase 2 (ASAH2), inhibiting the accumulation of lipid ROS 214. Upon ferroptosis, PMN-MDSCs release mediators such as oxidized phospholipids and PGE_2_, which strongly suppress CD8⁺ T cell function 212, 215. In certain models, such as glioblastoma, infiltrating neutrophils can conversely promote ferroptosis in tumor cells 216.

##### DCs

As professional APCs, DCs are highly susceptible to functional impairment by ferroptosis. Ferroptosis severely disrupts DCs maturation, manifested by downregulated expression of co-stimulatory molecules (CD80, CD86, CD40) and major histocompatibility complex class II (MHC II) molecules, as well as reduced secretion of key cytokines including IL-12, ultimately compromising their capacity for antigen presentation and naive T cell activation 217. Notably, the impact of ferroptosis on DCs function is phase-dependent: early ferroptotic tumor cells release DAMPs such as ATP to promote DCs maturation and elicit anti-tumor immunity, whereas late ferroptotic cells lose this immunogenicity and may even cause DCs dysfunction upon phagocytosis 13.

##### Natural killer (NK) cells

NK cells in the TME frequently display ferroptosis-associated hallmarks, including elevated lipid peroxidation and oxidative damage 218. Such metabolic disturbances suppress glucose metabolism in NK cells, impairing their cytotoxicity and production of cytokines (e.g., IFN-γ), leading to the exhaustion of their anti-tumor functions 219. Activation of the antioxidant transcription factor NRF2 can reverse this metabolic defect and restore NK cells activity 220. Furthermore, follistatin-like protein 1 secreted by CAFs activates p38 phosphorylation signaling in NK cells, upregulating NCOA4-mediated ferritinophagy, which results in cellular iron overload and ferroptosis induction 221. Therefore, protecting NK cells from ferroptosis is essential for maintaining their immune surveillance function.

#### 4.3.2 Adaptive Immune Cells

##### CD8⁺ Cytotoxic T Lymphocytes

CTLs exhibit a dual relationship with ferroptosis-mediated tumor immunity. On the one hand, activated CTLs serve as pivotal effector cells for inducing ferroptosis in tumor cells: the IFN-γ they secrete can significantly downregulate the expression of System xc⁻ transporter components (SLC7A11/SLC3A2) in tumor cells and simultaneously upregulate ACSL4 via activation of the JAK/STAT1 phosphorylation signaling pathway. In synergy with arachidonic acid in the TME, these events efficiently trigger ferroptosis in tumor cells 181. On the other hand, CTLs themselves are vulnerable to ferroptosis within the TME. A lipid-rich microenvironment induces high expression of the fatty acid transporter CD36 in CTLs. Uptake of oxidized low-density lipoprotein via CD36 activates the ASK1-p38 phosphorylation stress pathway, leading to the accumulation of lipid peroxidation products and subsequent T-cell senescence and functional exhaustion 200, 222, 223. Furthermore, programmed cell death protein 1 (PD-1) signaling enhances the susceptibility of CTLs to ferroptosis by inducing GATA-binding factor 1 to bind and repress phospholipid phosphatase 1 gene expression, thereby reducing phospholipid phosphatase 1 levels 224. Therefore, balancing the induction of ferroptosis in tumor cells and the protection of CTLs from ferroptosis represents a core challenge in the design of antitumor therapeutic strategies.

##### Regulatory T Cells (Tregs)

Tregs constitute a major cellular mediator of immunosuppression, while their ferroptosis resistance is highly reliant upon metabolic stability. Compared with effector T cells, Tregs are more tolerant to oxidative stress and ferroptosis, which is a feature linked in part to the robust upregulation of GPX4 expression by Tregs after receiving activating signals through their own T-cell receptor (TCR)/CD28 costimulatory pathways (involving complex phosphorylation cascades) 225. Targeted inactivation of GPX4 in Tregs triggers ferroptosis, with a consequent reduction in their immunosuppressive capacity, and indirectly increases tumor immunity through release of cytokines like IL-1β 226. These data imply that the fine-tuning of ubiquitination and degradation of master antioxidant enzymes in Tregs or their upstream signaling pathways may constitute a viable strategy to abolish TME-mediated immune suppression.

##### B Lymphocytes

Remarkable B-lymphocyte subset-specific differences exist in ferroptosis sensitivity. Because of their high expression levels of CD36 for fatty acid uptake, B1 cells and marginal zone B cells are strongly reliant on GPX4 to combat lipid peroxidation-induced cell damage. Notably, in this process, acyl-protein thioesterase 1-mediated depalmitoylation plays an essential role in regulating the formation of CD36/spleen tyrosine kinase-dependent signaling complexes that ultimately lead to prominent lipid peroxidation 24. In contrast, follicular B2 cells are relatively resistant to ferroptosis. They assemble a dual antioxidant defense system when IL-10 triggers activation of the STAT3 phosphorylation signaling pathway, maintaining the stability of the reduced GSH pool and inhibiting the activity of NADPH oxidase 2 227. Additionally, in autoimmune diseases such as systemic lupus erythematosus, B-cell ferroptosis has been verified to promote their differentiation into plasma cells and increase autoantibody production. However, in the tumor context, targeted induction of ferroptosis in regulatory B cells through PTM strategies to restore host antitumor immunity remains an urgent research area to be explored 228, 229.

### 4.4 Ferroptosis and Tumor Immune Escape

Ferroptosis not only directly kills tumor cells but also profoundly influences the efficacy of antitumor immune responses by regulating the functional status of immune cells within the TME, thereby participating in tumor immune escape at multiple levels.

On the one hand, tumor cells can achieve immune escape by enhancing their intrinsic resistance to ferroptosis. Several oncogenes (e.g., mutant RAS) and pathways (e.g., phosphatidylinositol 3-kinase [PI3K]-AKT-mammalian target of rapamycin complex 1) can enhance GSH synthesis by curbing the ubiquitination and degradation of SLC7A11, thus allowing escape from ferroptosis 230-232. AKT-induced phosphorylation of CKB also stabilizes GPX4 and strengthens the antioxidant response 100. Moreover, tumor cells undergo various metabolic reprogramming events to develop a ferroptotic-resistant phenotype (such as activating transcription factors like NRF2), leading to elevated synthesis of monounsaturated fatty acid-containing phospholipids and lower intracellular LIP levels, thus diminishing their sensitivity toward lipid peroxidation. This phenotype is highly correlated with tumor progression, cancer stemness maintenance, and distant metastasis 27, 43, 67, 233, 234.

On the other hand, the survival and functional stability of immunosuppressive cells in the TME (e.g., Tregs and MDSCs) depend on their resistance to ferroptosis. For example, Tregs highly express GPX4 to preserve lipid redox homeostasis and avoid ferroptotic cell death, thereby sustaining their immunosuppressive function 235. In MDSCs, high expression of ASAH2 confers ferroptosis resistance by attenuating lipid peroxidation and enhancing p53 protein stability, maintaining MDSC population size and suppressing CD8⁺ T cell activity 214. Based on this mechanism, targeting carnitine palmitoyltransferase 1A (CPT1A) can effectively induce ferroptosis in MDSCs and subsequently reverse the immunosuppressive state of the TME 236.

Furthermore, lipid peroxidation products (e.g., oxidized phospholipids) and certain mediators (e.g., PGE_2_) generated during ferroptosis can directly impair the viability and function of effector immune cells including T cells and NK cells, or indirectly shape an immunosuppressive microenvironment 29, 237, 238. Several long non-coding RNAs (e.g., ALMS1-IT1, OTUD6B-AS1) can upregulate SLC7A11 expression by recruiting and promoting STAT3 phosphorylation, establishing a synergistic interplay between ferroptosis resistance and immune escape 239, 240. The mitochondrial translocator protein TSPO and other molecules also inhibit ferroptosis via the NRF2 signaling pathway and upregulate PD-L1 transcription, further consolidating the molecular barriers of tumor immune escape 233.

A multi-layered, bidirectional regulatory network connects ferroptosis and tumor immune escape. Figure 5 systematically delineates the detailed processes through which ferroptosis mediates tumor immune escape via multiple routes: enhancing ferroptosis resistance in tumor cells, sustaining the function of immunosuppressive cells, and impairing the activity of effector immune cells. Tumor cells survive and proliferate by acquiring ferroptosis resistance, while immunosuppressive cells in the TME maintain their inhibitory function by resisting ferroptosis; these two events coordinately form the cellular barrier of tumor immune escape. Therefore, in-depth dissection of the precise regulatory mechanisms governing ferroptosis within the TIME and the development of combinatorial therapeutic strategies that selectively trigger ferroptosis in immunosuppressive cells while preserving effector immune cells will open a highly promising new avenue for overcoming resistance to tumor immunotherapy.

## 5. Roles of Protein PTM During Ferroptosis in Cancer Immunotherapy

By regulating core ferroptosis-related proteins, PTM play critical roles in cancer immunotherapy, with two primary functions: disrupting tumor immune evasion and activating the antitumor activity of effector immune cells. Figure 6 illustrates the molecular mechanisms and pathways through which PTM drive immune activation and reverse tumor immune evasion.

### 5.1 Roles of Ferroptosis-Related PTM in Disrupting Immune Evasion

Tumor immune evasion is a major hurdle for cancer initiation, progression and resistance to immunotherapy. Targeting ferroptosis-associated PTM has the potential to overcome this barrier through two approaches: increasing the immunogenicity of tumor cells and promoting the antigen presentation process, and directly modulating immune checkpoint molecule expression and function to abrogate their immunosuppressive signaling.

#### 5.1.1 Enhancing Tumor Antigen Expression and Presentation to Improve Immune Recognition

Tumor cell immunogenicity depends on surface antigen expression and antigen presentation efficiency. By tuning the activity of key ferroptotic proteins and antigen presentation molecules, PTM markedly strengthen the immune system's ability to recognize tumor cells.

##### Induction of ICD and Release of DAMPs

As stated earlier, ferroptosis has been confirmed as an immunogenic mode of cell death. When tumor cells undergo ferroptosis, they release substantial amounts of DAMPs, such as HMGB1 and ATP. These signals can be recognized by APCs including DCs, thereby triggering the host adaptive immune response 206. PTM directly influence the magnitude of ICD by regulating the susceptibility of tumor cells to ferroptosis. For instance, as detailed in Section 3.1.4, the deubiquitinase OTUD1 regulates iron homeostasis by stabilizing IREB2, thereby promoting ferroptosis in colorectal cancer cells, increasing DAMPs release, and enhancing immune cell cytotoxicity against cancer cells 94. This study indicates that targeting specific PTM-associated enzymes to induce ferroptosis in tumor cells may serve as an in situ vaccine strategy to effectively boost the host anti-tumor immune response.

##### Regulation of MHC Molecule Expression and Function

MHC I molecules present endogenous tumor antigens to CD8⁺ T cells, and reduced MHC I expression is a common mechanism underlying tumor immune escape 241. Ubiquitination and other PTM regulate tumor antigen presentation by controlling MHC I assembly, intracellular trafficking and cell surface stability 241. Evidence shows that tumor cells dysregulate ubiquitinase activity, causing abnormal MHC I ubiquitination. This leads to MHC I internalization, degradation or dysfunction, and eventually impairs antigen presentation 241, 242. Conversely, inducing ferroptosis in tumor cells may indirectly regulate the activity of transcription factors or ubiquitinating modifying enzymes involved in MHC class I expression by altering intracellular redox status and stress levels. For example, treatment of tumor cells with the ferroptosis inducers erastin or RSL3 can trigger a burst of intracellular ROS and lipid peroxidation 243, 244. Such robust cellular stress signals may stabilize or upregulate MHC class I molecule expression by activating the unfolded protein response or interfering with the function of the ubiquitin-proteasome system. Moreover, oxidized lipid components released during ferroptosis may serve as natural signaling molecules 245, either directly or indirectly affecting the activation of the antigen processing-related proteasome (immunoproteasome), thereby altering the configuration of intracellular antigen peptide repertoire and giving rise to neoantigens with great immunogenicity. While direct investigation in this area is still nascent, the potential relevance of PTM as a molecular link between ferroptotic stress and the antigen presentation process could be worth further study.

##### Regulation of Tumor-Associated Antigen and Neoantigen Expression

Ferroptosis-related PTM modulate transcription factor activities that are responsible for tumor-associated antigens (carcinoembryonic antigens, differentiation antigens, etc.) expression levels. One such transcription factor is STAT3 that is modified by a number of PTM, including acetylation and phosphorylation. It can not only prevent ferroptosis by enhancing the expression of GPX4 and SLC7A11, but also modulate the expression of a variety of immune-related genes 246. Inhibitors targeting acetylation-mediated activation of STAT3 (e.g., lysine acetyltransferase 6B inhibitors) may realize the dual effects of inducing ferroptosis in tumor cells and reconstructing the antigen landscape on tumor cells, thereby improving the immunogenicity of tumor cells 114.

#### 5.1.2 Modulating Immune Checkpoint Molecule Function to Block Immune Evasion Signals

Immune checkpoint molecules (e.g., PD-1/PD-L1, cytotoxic T-lymphocyte-associated protein 4 [CTLA-4]) represent a core molecular mechanism through which tumor cells suppress T-cell function and achieve immune evasion 247. Their membrane expression levels, endocytic recycling, and degradation processes are tightly regulated by PTM, especially ubiquitination.

##### Ubiquitination-Mediated Degradation of PD-L1

Cell surface PD-L1 levels directly determine its immunosuppressive activity 248. PD-L1 stability is controlled by two opposing enzyme families: E3 ubiquitin ligases (e.g., SPOP, CBL) and deubiquitinases (e.g., COP9 signalosome subunit 5). These factors target PD-L1 for degradation via the proteasomal or lysosomal pathway 249, 250. Multiple ferroptosis inducers and cellular stresses enhance PD-L1 ubiquitination and degradation by activating E3 ligases or suppressing deubiquitinases. For example, it has been shown that the deubiquitinase USP8 exerts its effects by stabilizing GPX4 and inhibiting ferroptosis, and therefore inhibition of USP8 leads to GPX4 instability and promotion of ferroptosis in tumor cells as well as improved immunotherapeutic efficacy by regulating its downstream substrates like PD-L1 251. METTL3 ubiquitination and degradation mediated by TRIM21 stimulates ferroptosis through inhibiting the expression of SLC7A11, and at the same time promotes PD-L1 expression in pancreatic cancer 252. This rather paradoxical observation suggests a complex feedback regulatory circuit: ferroptosis activates cytokine secretion including IFN-γ via the release of immunostimulatory signals, which subsequently feeds back to upregulate PD-L1 expression and forms a compensatory immune resistance pathway. In this context, synergistic anti-tumor effects can be achieved by combined treatment with PD-L1/PD-1 blockade, which will efficiently abrogate this compensatory inhibition 252. Together, these results uncover a dynamic and context-dependent interplay between PTM-regulated ferroptosis and immune checkpoint expression.

##### Regulation of Other Immune Checkpoint Molecules

Ubiquitination also regulates the expression of CTLA-4, thereby controlling its retention time on T-cell surface. The oxidative stress caused by ferroptosis or the altered enzyme activity of certain PTM may play an indirect role in regulating CTLA-4 ubiquitination and recycling processes 253, 254. Moreover, The functions of other emerging immune checkpoint molecules, including T-cell immunoglobulin and mucin-domain containing-3 and lymphocyte-activation gene 3, may also be regulated via PTM-dependent processes 255.

##### Indirect Regulation of Checkpoint Expression via Metabolic Reprogramming

Ferroptosis is closely intertwined with cellular metabolism, particularly lipid metabolism and glutamine metabolism. PTM can reshape the metabolite profile within the TME by modulating metabolic enzyme activity, thereby indirectly influencing immune checkpoint molecule expression. For example, FASN stabilizes GPX4 and inhibits ferroptosis by promoting the palmitoylation of USP5 15. Inhibiting FASN not only induces ferroptosis in tumor cells but may also attenuate immunosuppressive signals by altering the lipid composition of tumor cells and the profile of released metabolites, thereby remodeling the TME into a microenvironment more permissive for immune cell-mediated tumor attack 15.

### 5.2 Roles of PTM During Ferroptosis in Activating Immune Cells

Effective anti-tumor immune responses rely on the adequate activation, proliferation, infiltration and normal execution of cytotoxic functions of effector immune cells including CTLs and NK cells 256, 257. As previously elaborated, ferroptosis can significantly modulate the status and function of immune cells. This section focuses on the regulatory effects of ferroptosis-associated PTM on such effector immune cells.

#### 5.2.1 Promoting the Activation, Proliferation and Function of T Cells

##### Enhancing the Activation and Anti-Tumor Efficacy of CD8⁺ T Cells

CD8⁺ T cells serve as the core effector cells of anti-tumor immune responses. Studies have verified that inducing ferroptosis in tumor cells can markedly enhance the activity of CD8⁺ T cells 214, 258, and part of its regulatory mechanism relies on the precise control of key signaling pathways by PTM. Specifically, the CD8⁺ T cell-secreted IFN-γ can negatively regulate SLC7A11 and SLC3A2 expression on tumor cells, promoting ferroptosis in these cells 181. This is mediated by the JAK-STAT signaling pathway, and the activity of STAT proteins is tightly regulated through phosphorylation and dephosphorylation modifications 259, 260. Within TME, oxidized phospholipids such as SAPE-OOH which are released by ferroptotic cells, can function as "eat-me" signals recognized by Toll-like receptor 2 on the macrophage surface to facilitate phagocytosis of dead tumor cells and antigen presentation by macrophages and indirectly enhance T-cell activation 261. Furthermore, PTM can exert direct modulation on TCR signaling complex function. As an example, the intracellular ubiquitination state of critical proteins in the TCR signaling network (e.g., Zeta-chain-associated protein kinase 70, lymphocyte-specific protein tyrosine kinase) directly determines the strength of signal transduction and therefore impacts progression through T-cell activation 262, 263. Despite no direct experimental evidence supporting this hypothesis, Ferroptosis could modulate the PTM status of these kinases in T cells through changes to the redox potential or specific metabolite concentrations in the TME.

##### Sustaining the Long-Term Survival of Memory T Cells

The long-term persistence of memory CD4⁺ T cells is critical for the host to acquire sustained anti-tumor immune protection. Studies have shown that mammalian target of rapamycin complex 2 (mTORC2) can inhibit ferroptosis in virus-specific memory CD4⁺ T cells by phosphorylating downstream AKT and glycogen synthase kinase 3 beta, thereby promoting their long-term survival. This process involves the suppression of mitochondrial ROS accumulation and lipid peroxidation 264. The activity of mTORC2 and the phosphorylation status of its substrates represent a typical paradigm of PTM-mediated regulation. Therefore, targeting this PTM regulatory pathway may hold potential application value in enhancing memory immune responses following tumor vaccination.

#### 5.2.2 Enhancing the Cytotoxic Function of NK Cells

##### Modulating Activating and Inhibitory Receptors of NK Cells

The functional status of NK cells depends on the signaling balance between activating receptors (e.g., NK group 2D [NKG2D], DNAX accessory molecule-1) and inhibitory receptors (e.g., killer-cell immunoglobulin-like receptors, NKG2A) on their surface 265, 266. The membrane expression levels and intracellular recycling of these receptors are finely regulated by PTM such as ubiquitination. Ferroptosis can enhance the recognition capacity of NK cells against tumor cells by modulating the expression of ligands on the tumor cell surface, such as upregulating the NKG2D ligands MHC class I polypeptide-related sequence A/B. A direct regulatory cascade has emerged: intratumoral Brevibacillus parabrevis induces RAR-related orphan receptor C (RORC) acetylation, elevating NEDD4L expression. As noted in Section 3.1.4, NEDD4L targets iron transporters for degradation, restraining NK cell ferroptosis and potentiating anti-tumor immunity 267. This study suggests that specific PTM can stabilize the functional status of NK cells through cascade regulatory reactions, providing novel regulatory targets for enhancing NK cell-mediated anti-tumor immunity.

##### Preventing NK Cells Exhaustion

Similar to T cells, sustained activating stimulation and TME stress can also lead to functional exhaustion of NK cells, and ferroptosis may represent one of the mechanisms mediating NK cell exhaustion. Protecting NK cells from ferroptosis via PTM-related strategies (e.g., maintaining the protein stability of GPX4 or FSP1) may help preserve the durable tumor-killing capacity of NK cells, offering a new strategic direction for improving the efficacy of tumor immunotherapy.

#### 5.2.3 Promoting the Infiltration and Migration of Immune Cells into Tumor Tissues

##### Regulating Chemokines and Their Receptors

Directed homing and infiltration of immune cells at tumor sites are tightly controlled primarily by the chemokine-receptor axis. PTM process this in a two-fold manner: firstly, they modulate the types and amounts of chemokines secreted by either tumor cells or stromal cells; second, they regulate the expression levels and functional activity of chemokine receptors on immune cell membranes. For example, certain lipid mediators or DAMPs released during ferroptosis can act as potent chemotactic signals, effectively recruiting DCs and macrophages to infiltrate the tumor. Furthermore, studies have demonstrated that inhibiting the deubiquitinase USP52 can promote the infiltration of CD8⁺ T cells into tumor tissues by stabilizing the YAP protein 268. Although USP52 mainly functions to suppress ferroptosis in colorectal cancer cells, its regulated YAP target genes may include factors involved in extracellular matrix remodeling and immune cell recruitment 268, indicating that it can achieve coordinated regulation of ferroptosis and immune cell infiltration via PTM modulation.

##### Ameliorating the Immunosuppressive Microenvironment

Ferroptosis can indirectly create favorable conditions for the infiltration of effector immune cells by eliminating immunosuppressive cells (e.g., M2-type TAMs, Tregs) or regulating their functional status, and PTM play a key regulatory role in this cellular functional transformation process. For instance, BEBT-908, a dual PI3K/HDAC inhibitor, can upregulate the expression of MHC class I molecules on the tumor cell surface and activate the STAT1/IFN-γ signaling pathway by inducing ferroptosis in tumor cells, thereby boosting anti-tumor immune responses 269. Among these, HDAC inhibitors can simultaneously regulate the expression of ferroptosis-related genes and immune-related genes by altering the acetylation status of histones and non-histone proteins 269, forming a PTM-mediated synergistic effect between ferroptosis and immune microenvironment improvement.

### 5.3 Other Roles of PTM During Ferroptosis in Cancer Immunotherapy

Besides directly interfering with tumor immune evasion and activating effector immune cells, ferroptosis-associated PTM can also drastically modulate the effectiveness of cancer immunotherapy by more widespread regulatory methods, like modulation of tumor metabolism, the intratumoral microbiome, and intercellular communication.

#### 5.3.1 Modulating the Crosstalk Between Tumor Metabolism and Immunometabolism

Tumor cells and immune cells compete for finite nutritional resources within the TME. Interestingly, PTM play a critical role in regulating the activity of metabolic enzymes and ferroptosis is closely linked to many metabolic pathways including GSH synthesis, lipid oxidation and iron metabolism.

##### Targeting Amino Acid Metabolism

SLC7A11 acts as a core negative regulator of ferroptosis, and its function is modulated by diverse PTM, as detailed in Sections 3.1.1, 3.2.1 and 3.5 150, 270, 271. Inhibition of SLC7A11 not only triggers ferroptosis in tumor cells but also indirectly regulates T-cell function by depleting cysteine and elevating glutamate release in the TME. For instance, IFN-γ secreted by CD8⁺ T cells can suppress SLC7A11 expression, inducing cysteine starvation and ferroptosis in tumor cells. Cysteinase treatment mimics this effect and exerts synergistic anti-tumor activity with anti-PD-L1 antibodies 181, 272. Therefore, targeting modifying enzymes associated with SLC7A11-related PTM represents a novel metabolic intervention strategy, which can directly eliminate tumor cells while remodeling the metabolic microenvironment for T cells to enhance anti-tumor immune responses.

##### Targeting Lipid Metabolism

Lipid peroxidation constitutes the core execution step of ferroptosis. As the rate-limiting enzyme of fatty acid oxidation, CPT1A forms a positive feedback loop with cellular myelocytomatosis oncogene to activate the NRF2/GPX4 signaling pathway and downregulate ACSL4 expression, thereby suppressing ferroptosis in lung cancer stem cells and inactivating CD8⁺ T cells 236. Targeting CPT1A reverses these processes and markedly improves the therapeutic efficacy of immune checkpoint blockade. Likewise, as previously described in Section 3.5, FASN-driven palmitoylation sustains USP5 activity, preserving GPX4 stability and suppressing ferroptosis in breast cancer cells to blunt immunotherapy efficacy 15. As documented in Section 3.2.2, PKCβII-mediated ACSL4 phosphorylation enhances lipid peroxidation and ferroptosis, creating synergy with immunotherapy 102. These findings illustrate how PTM-induced modulation of the activity of lipid metabolic enzymes enables coordinated regulation of ferroptosis sensitivity in tumor cells and immune cell function, opening avenues for optimizing cancer immunotherapy approaches.

#### 5.3.2 Regulating the Crosstalk Between the Intratumoral Microbiome and Immunity

Recent studies confirmed that intratumorally colonized microorganisms modulate local tumor immune processes via several mechanisms. In hepatocellular carcinoma, it was found that colonization of tumors with Brevibacillus parabrevis triggers a signaling cascade downstream by catalyzing the acetylation of RORC, which ultimately inhibits ferroptosis in NK cells via NEDD4L-mediated ubiquitination and degradation of iron transporters, enhancing their anti-tumor immune activity 267. This study reveals a new regulatory mechanism wherein intratumoral microbes can remotely control key cell death pathways in immune cells through modulating the host PTM system, thus actively shaping the anti-TIME on-site. This finding provides insights into the potential application of microorganisms or their metabolites as biological tools to modulate PTM and ferroptosis, which may improve cancer immunotherapy approaches.

#### 5.3.3 Mediating the Propagation Effect of Ferroptosis

Apoptotic cell death is usually confined to the primary injured cells, while ferroptosis has a special spreading feature that ferroptosis in one cell can trigger adjacent cell poisoning 273. In relevant studies, ferroptotic cells secrete galectin-13, which acts upon the CD44 molecule of nearby cells and inhibits membrane localization of SLC7A11, thereby driving neighboring cells into ferroptosis 274. The initiation of this propagation process involves the phosphorylation of forkhead box K1 (FOXK1) by PKCβII 274. This PTM-mediated paracrine signaling can significantly amplify the anticancer effects of ferroptosis, exerting particularly potent killing effects on cancer stem cells, and acts synergistically with radiotherapy and immunotherapy, providing novel targets for improving the efficacy of tumor therapy.

#### 5.3.4 Providing Novel Combination Therapeutic Strategies and Biomarkers

The development of specific inhibitors or activators targeting key PTM sites and related enzymes in the ferroptosis regulatory network has become an emerging research direction for sensitizing tumor immunotherapy. To date, molecules including USP8, USP52, FASN, CPT1A, PKCβII, and TRIM21 have been validated as critical hubs that coordinately regulate ferroptosis and immune responses 15, 102, 236, 251, 252, 268. Their corresponding small-molecule modulators are promising as next-generation combination agents for immunotherapy, enhancing clinical therapeutic efficacy by remodeling ferroptosis sensitivity in the TME. On this basis, proteolysis-targeting chimera (PROTAC) technology, which targets the degradation of core anti-ferroptotic proteins such as GPX4, offers a cutting-edge strategy for highly specific ferroptosis induction. If such degraders can achieve selective activation within tumor tissues, they will exert robust synergistic antitumor effects with ICIs 275, 276. In addition, the realization of precision medicine relies on the identification of PTM-related biomarkers to effectively predict immunotherapy responses. For instance, DL receptor-related protein 1B gene mutations can modulate STAT3 phosphorylation levels, thereby affecting SLC7A11 expression and ferroptosis sensitivity in tumor cells, representing a potential predictive marker for immunotherapy benefits in patients with lung adenocarcinoma 277. Conversely, high expression of Cytochrome P450 1B1 induces ferroptosis resistance in tumor cells via FBXO10-mediated ubiquitination and degradation of ACSL4, leading to resistance to anti-PD-1 therapy in patients with colorectal cancer 278. In the future, these PTM-associated molecules and their dynamic modification status are expected to serve as important biomarkers for selecting immunotherapy-responsive patient populations and real-time monitoring of therapeutic efficacy.

#### 5.3.5 Potential Risks and Opportunities Affecting Immune-Related Adverse Events

Notably, PTM modulators (e.g., HDAC inhibitors, proteasome inhibitors) are frequently associated with off-target effects and related adverse reactions in clinical practice, such as myelosuppression and neurotoxicity 279, 280. This indicates that when designing ferroptosis-immunotherapy combination strategies targeting PTM, the selectivity of regulation must be prioritized to avoid excessive damage to normal tissues, particularly the immune system itself. Nevertheless, under certain conditions, such broad regulatory effects can be rationally harnessed and converted into therapeutic advantages. For example, the antiplatelet drug vorapaxar has been found to upregulate heme oxygenase 1 expression by binding to forkhead box O1 and inhibiting its phosphorylation, inducing mitochondrial iron overload and ferroptosis, thereby enhancing immunotherapeutic efficacy. Meanwhile, its intrinsic antithrombotic activity mitigates the high risk of thrombosis commonly observed in cancer patients 281, achieving dual benefits of therapeutic efficacy and complication control.

### 5.4 Critical Synthesis and Context-Dependent Nuances of PTM-Modulated Ferroptosis in Immunotherapy

The above sections delineate a complex, non-linear regulatory landscape where protein PTM act as molecular rheostats that calibrate ferroptosis to shape immunotherapy responses—not as unidirectional switches, but as context-dependent modifiers with profound therapeutic trade-offs. No single PTM-ferroptosis axis uniformly promotes anti-tumor immunity; instead, their effects are contingent on PTM type, target protein identity, cellular compartment, and microenvironmental context, creating critical nuances that underpin both therapeutic opportunities and translational challenges.

First, ubiquitination-mediated ferroptosis regulation exhibits inherent duality in immune checkpoint control, a key unresolved paradox in this field. As documented, deubiquitinases such as USP8 stabilize GPX4 to suppress ferroptosis while concurrently sustaining PD-L1 expression, linking ferroptosis resistance directly to immune evasion 251. Conversely, TRIM21-driven METTL3 ubiquitination promotes ferroptosis but paradoxically upregulates PD-L1 in pancreatic cancer, revealing a compensatory immune resistance loop where ferroptosis-induced inflammation feeds back to activate checkpoint signaling 252. This contradiction underscores a critical caveat: targeting ferroptosis alone may not suffice to abrogate immune evasion, as PTM-coupled feedback circuits can counteract immunostimulatory effects—a nuance largely overlooked in simplified ferroptosis-immune models.

Second, acetylation and phosphorylation of core transcription factors and metabolic enzymes drive context-dependent immune-metabolic crosstalk, with profound implications for immunotherapy efficacy. STAT3 acetylation, for instance, exerts dual roles: it suppresses ferroptosis via GPX4/SLC7A11 upregulation while remodeling the tumor antigen landscape, creating a trade-off between ferroptosis resistance and immunogenicity 114. Similarly, PKCβII-mediated ACSL4 phosphorylation enhances lipid peroxidation and ferroptosis, yet its downstream effects on immunity depend on the tumor's metabolic state—high lipid TME amplify ferroptotic immunogenicity, while glutamine-depleted TME blunt it 102. These observations challenge the notion of “one-size-fits-all” PTM-targeted ferroptosis induction, highlighting that metabolic context dictates whether a given PTM event enhances or diminishes immunotherapy responses.

Third, cell-type-specific PTM regulation of ferroptosis creates a delicate balance between eliminating tumor cells and preserving anti-tumor immunity, a critical consideration for therapeutic design. In tumor cells, PTM (e.g., FASN-driven palmitoylation of USP5) stabilize GPX4 to confer ferroptosis resistance and immune evasion 15. In contrast, in NK cells, NEDD4L-mediated ubiquitination of iron transporters restrains ferroptosis and preserves cytotoxicity, while mTORC2-dependent phosphorylation sustains memory T cell survival 264, 267. This cell-type dichotomy implies that systemic PTM modulation risks off-target ferroptosis in effector immune cells, blunting immunotherapy efficacy—a key reason why many pan-ferroptosis inducers fail in combination with ICIs. Targeting tumor-specific PTM-ferroptosis nodes (e.g., USP8 inhibition in GPX4-high tumors) is therefore essential to avoid compromising immune cell function.

Fourth, non-canonical PTM-ferroptosis axes—including intratumoral microbiome signaling and ferroptosis propagation—add layers of complexity to immunotherapy outcomes. Brevibacillus parabrevis-induced RORC acetylation inhibits NK cell ferroptosis and enhances immunity, representing a microbe-host PTM crosstalk that can be harnessed therapeutically 267. However, galectin-13-mediated ferroptosis propagation, driven by PKCβII-dependent FOXK1 phosphorylation, exerts dual effects: it amplifies tumor cell killing but also triggers widespread immunosuppressive lipid release in dense TME, limiting its utility in advanced tumors 274. These findings illustrate that non-tumor-derived PTM signals can reshape ferroptosis-immune crosstalk, introducing both opportunities and unforeseen challenges for combinatorial immunotherapy.

Collectively, PTM-modulated ferroptosis is not a linear driver of immunotherapy response but a dynamic, context-dependent regulatory hub defined by dualities, trade-offs, and cell-type specificity. Simplified models that ignore PTM heterogeneity and microenvironmental context risk misguiding therapeutic development. Instead, future strategies must prioritize precision PTM targeting—tailoring interventions to specific PTM events, tumor subtypes, and metabolic contexts—to amplify ferroptosis-driven immunostimulation while mitigating immunosuppressive feedback and immune cell toxicity. This nuanced perspective is critical to unlocking the full potential of ferroptosis-PTM targeted immunotherapy.

## 6. Application Prospects of Ferroptosis in Cancer Immunotherapy

### 6.1 Synergistic Therapy of Ferroptosis and ICIs

The core of combining ferroptosis with ICIs lies in restarting and amplifying the host's anti-tumor immune response via a positive feedback loop (detailed in Sections 4.1.3 and 4.3.2) 282, 283: ICIs reinvigorate CD8⁺ T cells, whose secreted IFN-γ enhances tumor ferroptosis sensitivity; in turn, immunogenic ferroptotic cell death promotes DC maturation and T cell priming, further strengthening anti-tumor immunity 11, 171, 284.

Based on the above biological basis, numerous preclinical studies have provided encouraging experimental evidence for the combination therapy of ferroptosis and ICIs. The combined application of various types of ferroptosis inducers targeting key ferroptotic pathways with ICIs has shown superior tumor suppression and survival benefits over monotherapy in multiple refractory tumor models 285-287. For example, inhibitors targeting System xc⁻ (e.g., the erastin derivative IKE, sulfasalazine) or direct GPX4 inhibitors (e.g., RSL3), when combined with anti-PD-1/PD-L1 antibodies, not only directly kill tumor cells but also significantly increase the infiltration of CD8⁺ T cells and M1-type macrophages in tumor tissues, while reducing the proportion of immunosuppressive cells such as Tregs and MDSCs, effectively remodeling the immune status of the TME 169, 288-291.

More promising for clinical translation is that many drugs already in clinical use or with favorable safety profiles have been rediscovered to possess ferroptosis-inducing activity, greatly accelerating the clinical exploration of combination therapeutic regimens. For instance, statins downregulate GPX4 expression by inhibiting the mevalonate pathway and can reduce PD-L1 expression levels in non-small cell lung cancer models, converting non-small cell lung cancer into an inflammatory phenotype sensitive to immunotherapy 292. The antimalarial drug mefloquine enhances the sensitivity of melanoma and lung cancer to PD-1 inhibitors by activating the IFN-γ-STAT1-interferon regulatory factor 1 signaling axis, upregulating LPCAT3 expression, and promoting lipid peroxidation 293. In addition, the application of nanotechnology provides innovative solutions to the targeted delivery, controlled release, and reduced systemic toxicity of ferroptosis inducers 294. Novel delivery systems such as injectable hydrogels loaded with sulfasalazine and metal-organic framework nanoparticles enable sustained local drug release in tumors, efficiently inducing ferroptosis in tumor cells while effectively activating local and systemic anti-tumor immune responses, and exerting robust synergistic anti-tumor effects with ICIs 289, 295. Collectively, these studies point to a future direction for cancer therapy: using ferroptosis induction as a powerful immunomodulatory switch in rational combination with existing ICIs represents one of the key strategies to break the current bottlenecks of immunotherapy and expand the population benefiting from cancer immunotherapy.

### 6.2 Development of Small-Molecule Drugs Targeting the Ferroptosis Pathway

The advancement of small molecule agents with high efficiency, specificity and excellent pharmacokinetic properties is essential for successful translation of ferroptosis regulatory strategies into clinical tumor therapeutic approaches. Currently, key pathways for drug development aimed at the core system of ferroptosis are being actively explored with their own unique clinical application prospects 296.

One pathway: Inhibitors of System xc⁻. Representative examples of this class of drugs include erastin and its improved derivative, IKE, as well as the clinically available anti-inflammatory sulfasalazine 297-299. The underlying therapeutic advantage associated with these agents is their established clinical indications, compared to a more complete safety profile and adequate preclinical rationale for combination 300. Future development directions mainly include: maximizing drug selectivity, improving the tumor-targeted delivery efficiency of immunosomes, and exploring optimal combinatorial regimens incorporating distinct ICIs to maximize clinical therapeutic benefits.

The second category comprises inhibitors that directly target GPX4. In turn, first-generation GPX4 inhibitors (e.g., RSL3, ML162) generally have low water solubility and fast in vivo metabolism and can harm normal tissues, which has greatly restricted their progress of clinical translation 301. The frontier of current studies in this area includes development of next-generation GPX4 inhibitors with improved pharmacokinetic properties and increased tumor-selectivity. For instance, prodrug modification strategies, nanocarrier-mediated targeted delivery, and TME-responsive activation enable precise drug release within tumors. Such approaches efficiently eliminate tumor cells while maximally preserving the function of normal immune cells 16, 302. In addition, the development of novel drug modalities such as PROTACs offers new strategies to selectively deplete GPX4 protein in tumor cells 303.

The third pathway: Inhibitors of alternative ferroptosis defense pathways. FSP1 inhibitors (e.g., iFSP1) disrupt CoQ10 regeneration at the plasma membrane, and are particularly suitable for GPX4 inhibitor-insensitive tumors with FSP1 compensatory overexpression, exerting synergistic effects with immunotherapy 304. DHODH inhibitors (e.g., brequinar) selectively induce ferroptosis in GPX4-low tumor cells via targeting the mitochondrial antioxidant pathway, demonstrating potential for biomarker-guided precision therapy 37.

Finally, small-molecule drugs targeting tumor metabolic vulnerabilities also show great clinical application potential. For example, targeting CPT1A can disrupt the fatty acid oxidation process and intracellular antioxidant balance of tumor cells, thereby inducing ferroptosis 236; inhibiting phosphoglycerate mutase 1 can downregulate lipocalin 2 through energy stress and ROS-dependent pathways, promote ferroptosis in hepatocellular carcinoma cells, and enhance the infiltration of CD8⁺ T cells into tumor tissues 285. Such drugs usually act on tumor cell-specific metabolic reprogramming nodes and may have a better therapeutic window compared with traditional chemotherapeutic drugs, reducing damage to normal tissues.

In general, the development of next-generation ferroptosis inducers will prioritize precision and tumor selectivity. Specifically, structural optimization of lead compounds, deployment of innovative targeted delivery platforms, and rational design of combinatorial regimens can enable tumor-specific targeting, maximally preserve host anti-tumor immune function, and lower systemic drug toxicity. The development of this field will become an innovative research frontier of interdisciplinary integration, providing important support for promoting the translation of ferroptosis therapy from basic research to clinical practice.

### 6.3 Therapeutic Strategies Focusing on PTM of Ferroptosis-Associated Proteins

In addition to small-molecule drugs targeting the core ferroptosis pathway, the specific regulation of PTM of ferroptosis-associated proteins has become an important direction for optimizing tumor immunotherapy strategies, which can achieve precise regulation of the ferroptosis pathway and reduce off-target effects. Among them, the targeted regulation of ubiquitination/deubiquitination modifications is one of the research hotspots. The deubiquitinase USP8 has been confirmed to stabilize GPX4 protein; inhibiting USP8 can disrupt the protein stability of GPX4, thereby enhancing the sensitivity of tumor cells to ferroptosis, and synergistically exerting tumor-suppressive effects with anti-PD-1 therapy in in vivo experiments 251. In addition, E3 ubiquitin ligases (e.g., TRIM25, TRIM21, etc.) have also been found to be involved in the regulation of SLC7A11 or GPX4 degradation. Targeting such specific E3 ubiquitin ligases or deubiquitinases can precisely regulate the activity of the ferroptosis pathway, avoiding off-target effects arising from non-selective pan-modulation of ferroptosis signaling 16, 252, and providing a safer strategy option for clinical treatment.

In addition to classic PTM types, novel modification methods such as protein hydroxylation have also added a new dimension to ferroptosis regulation. Studies have found that phosphoserine aminotransferase 1 (PSAT1) can undergo phosphorylation under IFN-γ stimulation, thereby promoting the hydroxylation of GPX4 protein at the P159 site by prolyl hydroxylase 3 151. This hydroxylation modification can block the binding of GPX4 to the chaperone HSC70, inhibit its degradation through the autophagic pathway, thereby stabilizing GPX4 protein expression and promoting tumor cell resistance to ferroptosis 151. Therefore, the development of small-molecule inhibitors that can interfere with PSAT1 phosphorylation or GPX4 hydroxylation is expected to specifically abrogate IFN-γ-induced ferroptosis resistance in tumor tissues, thereby exerting a strong synergistic anti-tumor effect with ICIs.

As an important epitranscriptomic regulatory mechanism, RNA m⁶A modification is also deeply involved in the regulation of ferroptosis-related gene expression 305. For example, the methyltransferase METTL3 can stabilize the expression of SLC7A11 mRNA through m⁶A modification, promote its translation process, and thereby inhibit tumor cell ferroptosis 252. The protein YTHDF1 may upregulate PD-L1 expression and inhibit ferroptosis through a similar m⁶A recognition and regulation mechanism, forming a dual barrier of tumor cell immune evasion and ferroptosis resistance 306. Based on this, the development of drugs targeting specific m⁶A modification enzymes (e.g., METTL3 inhibitors) or interfering with their recognition process is expected to simultaneously break the ferroptosis resistance and immunosuppressive state of tumor cells, achieve more efficient anti-tumor therapeutic effects, and provide a new research direction for the combination strategy of ferroptosis and immunotherapy.

### 6.4 Formulation of Personalized Treatment Regimens

Given the high heterogeneity of tumors and the complexity of the ferroptosis regulatory network, the future combined application of ferroptosis and immunotherapy will inevitably move toward individualization and precision. Identifying and validating biomarkers that can predict therapeutic effects is the core link to achieve this goal, as well as a current research focus and difficulty in this field. An ideal biomarker should comprehensively reflect the intrinsic ferroptosis sensitivity of tumor cells and the immune characteristics of the TME, providing precise guidance for the formulation of treatment regimens. Currently, biomarkers at multiple levels have shown potential clinical application prospects:

At the molecular level, low expression of SLC7A11 or GPX4, as well as high expression of ACSL4 and LPCAT3 in tumor tissues, usually indicates that tumor cells are more sensitive to ferroptosis induction; clinical data analysis shows that tumor patients with such molecular characteristics often have better therapeutic responses and survival benefits to ICIs 11, 169, 290. In contrast, high expression of FSP1 may indicate tumor cell resistance to GPX4 inhibitors, but such tumors may be sensitive to FSP1 inhibitors 304. Sustained activation of the NRF2 pathway caused by KEAP1 mutations is a well-recognized molecular event associated with resistance to radiotherapy, chemotherapy, and immunotherapy in various tumors such as lung cancer. The expression level of its key downstream factors (e.g., tumor-infiltrating lymphocytes [TILs], NAD(P)H quinone dehydrogenase 1 [NQO1]) may become a potential biomarker for guiding combination therapy (e.g., using NRF2/NQO1 axis inhibitors) 307.

At the cellular and pathological levels, the immune typing of tumors is an important basis for determining whether to combine ferroptosis inducers 308. For "cold tumors" lacking T cell infiltration, combined use of ferroptosis inducers to enhance tumor immunogenicity and recruit immune cell infiltration is a reasonable treatment strategy; for patients with established "hot tumors" but T cell dysfunction, it is necessary to carefully evaluate the potential damage of ferroptosis inducers to infiltrating immune cells, or select specific drugs with lower toxicity to immune cells 309, 310. Indicators such as the density and exhaustion status of TILs (especially CD8⁺ T cells), and the subtype ratio of MDSCs and TAMs can be quantitatively analyzed by technologies such as multiplex immunofluorescence, flow cytometry, or spatial transcriptomics, providing multi-dimensional reference information for the formulation of personalized treatment regimens 311.

At the systemic level, constructing multi-gene prediction models using high-throughput omics data is an important future development trend 312. By integrating transcriptome, proteome, and even metabolome data, a composite scoring systems that integrate the activity of ferroptosis-related pathways and tumor immune status can be established. For example, prognostic/predictive models based on ferroptosis-related long non-coding RNAs or ferroptosis-related genes have shown potential in distinguishing patient prognosis and predicting immunotherapeutic effects in tumor datasets such as hepatocellular carcinoma and renal cell carcinoma 313, 314. These digital scoring models are expected to be further verified and improved in subsequent clinical trials, and ultimately transformed into effective tools for guiding clinical decision-making.

Therefore, future clinical practice may follow the following path: through multi-omics analysis of patients' tumor samples, comprehensively evaluate the activity of ferroptosis-related pathways, the characteristics of the immune microenvironment, and key driver mutations, and classify patients into different molecular subtypes; for different subtypes, match the most appropriate type of ferroptosis inducer and specific ICIs drugs, and if necessary, combine with other treatment methods such as radiotherapy to form a personalized combination treatment regimen. At the same time, dynamically monitor relevant biomarkers in blood or imaging during the treatment process to realize real-time adjustment and optimization of the treatment regimen. This model marks that tumor immunotherapy will move from the traditional "one-size-fits-all" model to the era of truly precision medicine.

## 7. Conclusions and Future Perspectives

As a regulated cell death mode characterized by iron-dependent lipid peroxidation, ferroptosis has opened up a new research path for overcoming tumor apoptosis resistance 315. Scholars widely agree that PTM — mainly ubiquitination, phosphorylation and acetylation — form a highly dynamic, fine-tuned regulatory layer operating beyond gene transcription. By modulating the stability, enzymatic activity and subcellular localization of core ferroptosis regulators including SLC7A11, GPX4, FSP1 and ACSL4, PTM profoundly alter tumor cell fate and act as a critical molecular link between tumor metabolism and antitumor immunity 8, 34, 316. Current research has gradually shifted from the initial identification of ferroptosis regulators to the in-depth analysis of PTM regulatory codes, such as the dynamic regulation of GPX4 by E3 ubiquitin ligases and deubiquitinases 317, the bidirectional regulation of SLC7A11 or ACSL4 by kinases through site-specific phosphorylation 318, 319, and the regulatory role of deacetylases such as SIRT3 in maintaining mitochondrial oxidative balance 115. The in-depth integration of artificial intelligence, machine learning, and protein structure prediction technologies such as AlphaFold2 is revealing the regulatory effects of PTM modification status on protein function at the atomic level by integrating high-throughput omics data, accelerating the mapping of the global regulatory landscape of ferroptosis-related PTM 320-325.

To further improve the precision and translational potential of ferroptosis-targeted therapy, future studies may establish a practical technical framework integrating AI and structural biology. Specifically, AI algorithms coupled with high-resolution protein structure predictors such as AlphaFold2 can systematically model structural features and PTM sites of core ferroptosis-regulatory enzymes, such as E3 ubiquitin ligases and deubiquitinases. This approach supports targeted screening and rational design of highly selective small-molecule inhibitors against these enzymes. It offers a viable strategy to selectively trigger ferroptosis in tumor cells while protecting antitumor effector T cells from ferroptotic injury. This strategy is expected to overcome the non-specific cell killing bottleneck of current ferroptosis-based therapies and boost the translational value of related research.

At the tumor-immune interface, ferroptosis and immune responses interact in a complex dual fashion. Activated T cells trigger tumor cell ferroptosis via IFN-γ signaling and act synergistically with ICIs, while certain oxidized lipids generated during this process may suppress immune activity. This duality highlights the context dependence of ferroptosis-related therapeutic strategies 326, 327. Figure 7 illustrates the core strategies and development directions for optimizing the efficacy of tumor immunotherapy by regulating ferroptosis through PTM-based approaches in the future. Based on the above research foundation, the combined application of targeted PTM regulatory networks and immunotherapy shows broad clinical translation prospects. Inducing tumor cell ferroptosis and releasing DAMPs through small-molecule drugs to reverse immune resistance, while leveraging nanodelivery technologies and multi-omics prediction models, is expected to achieve precise and personalized combination therapy in the future 292, 328, 329.

Despite promising prospects, translating strategies targeting the PTM-ferroptosis axis into clinical therapies faces four major challenges (see Table 3 for corresponding translational strategies and existing hurdles) 74, 330:

First, the selective regulation of ferroptosis induction in cells is the primary problem to be solved. The current core bottleneck lies in how to accurately induce ferroptosis in tumor cells while effectively avoiding damage to anti-tumor effector immune cells such as CD8⁺ T cells. This requires researchers to use advanced technologies such as single-cell sequencing and spatial proteomics to deeply analyze the essential differences in PTM regulatory networks between tumor cells and immune cells, and explore PTM vulnerability sites that are highly activated only in malignant tumor cells, providing theoretical support for achieving precise targeting.

Second, the core determinant mechanism of ferroptosis immunogenicity needs further in-depth exploration. Future research should go beyond the macro observation of classical DAMPs release and focus on how different PTM modification patterns shape specific lipid peroxidation product profiles by regulating metabolic enzyme activity. For example, it is necessary to in-depth analyze how protein covalent adducts formed by lipid peroxidation by-products such as 4-HNE are recognized by immune receptors as specific signaling molecules, thereby determining the evolution of the immune system toward pro-inflammatory activation or immunosuppressive tolerance. This is crucial for optimizing the combination treatment regimen of ferroptosis and ICIs.

Third, the complexity and redundancy of the PTM regulatory network itself constitute a major challenge for clinical treatment. During the dynamic evolution of the TME, the same protein is often regulated by crosstalk between multiple PTM types. For example, phosphorylation modification may serve as a pre-signal to induce subsequent ubiquitination and degradation processes 331. The precision of this PTM regulatory code means that single-target intervention is likely to trigger compensatory signaling pathways in cells, ultimately leading to treatment resistance. Therefore, systematic analysis of the PTM interaction map and its real-time dynamic changes is of great theoretical and practical significance for preventing and overcoming clinical treatment resistance.

Fourth, research on the mechanisms of emerging PTM types and cross-level regulation represents a new frontier direction in this field. In addition to classical ubiquitination and phosphorylation modifications, there are still a large number of research gaps in how emerging PTM modifications such as lactylation and SUMOylation, as well as RNA modifications such as m⁶A, participate in ferroptosis regulation by regulating protein function or mRNA stability 140. In-depth exploration of these multi-dimensional regulatory landscapes is expected to reveal new ferroptosis regulatory targets, providing a solid theoretical basis for constructing more efficient and specific tumor immunotherapy strategies.

Collectively, the above research advances, existing translational bottlenecks, and future research directions jointly define the whole developmental process of ferroptosis-targeted cancer therapy. Figure 8 systematically summarizes the complete clinical translation trajectory of PTM-regulated ferroptosis therapy, covering the progressive phases from basic preclinical mechanism research, technological optimization and strategy improvement, translational verification to final personalized clinical application.

But these studies are systematically driving us to a novel realm of cellular network regulation: from linear pathway regulatory Darwinism towards an understanding of regulation mediated by dynamic protein and gene interactions during ferroptosis. However, the powerful programmability of cell death has also been utilized in biomedicine, as evidenced by recent advancements in tumor immunotherapy against a multitude of cancers. Despite numerous scientific mysteries and clinical translation challenges in the future, targeted intervention to this high-precision PTM regulatory network is expected to eventually reach the ultimate goal of selective clearance of tumor cells and efficient activation of long-term host anti-tumor immunity through deep interdisciplinary cooperation and technological innovation, leading cancer treatment into a new era by bringing fundamental breakthroughs and changes.

## Figures and Tables

**Figure 1:**
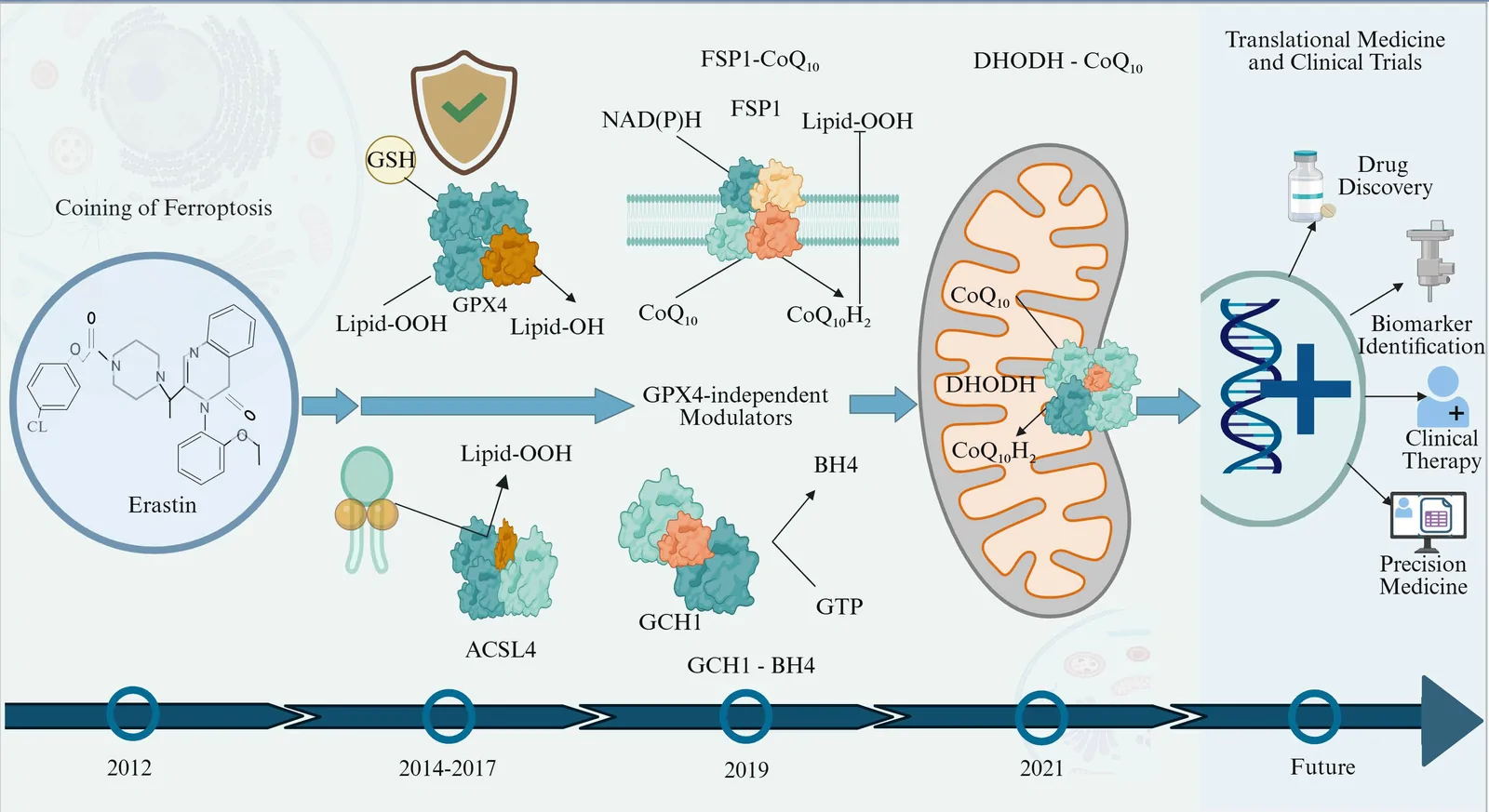
** Key milestones in the evolution of ferroptosis research.** The timeline highlights the formal coining of "ferroptosis" and the identification of erastin as a selective inducer in 2012. This was followed by the characterization of core regulatory machinery between 2014 and 2017, establishing GPX4 as the central inhibitor and ACSL4 as a critical pro-ferroptotic driver of lipid peroxidation. Recent breakthroughs (2019-2021) expanded the field through the discovery of GPX4-independent defense systems, including the FSP1-CoQ10, GCH1-BH4, and mitochondrial DHODH pathways. Current and future research directions focus on clinical translation, aiming to leverage ferroptosis for cancer immunotherapy and targeted drug applications. **GPX4:** Glutathione Peroxidase 4; **ACSL4:** Acyl-CoA synthetase long-chain family member 4; **FSP1:** ferroptosis suppressor protein 1; **CoQ10:** Coenzyme Q10; **GCH1:** GTP cyclohydrolase 1; **BH4:** Tetrahydrobiopterin; **DHODH:** Dihydroorotate dehydrogenase.

**Figure 2:**
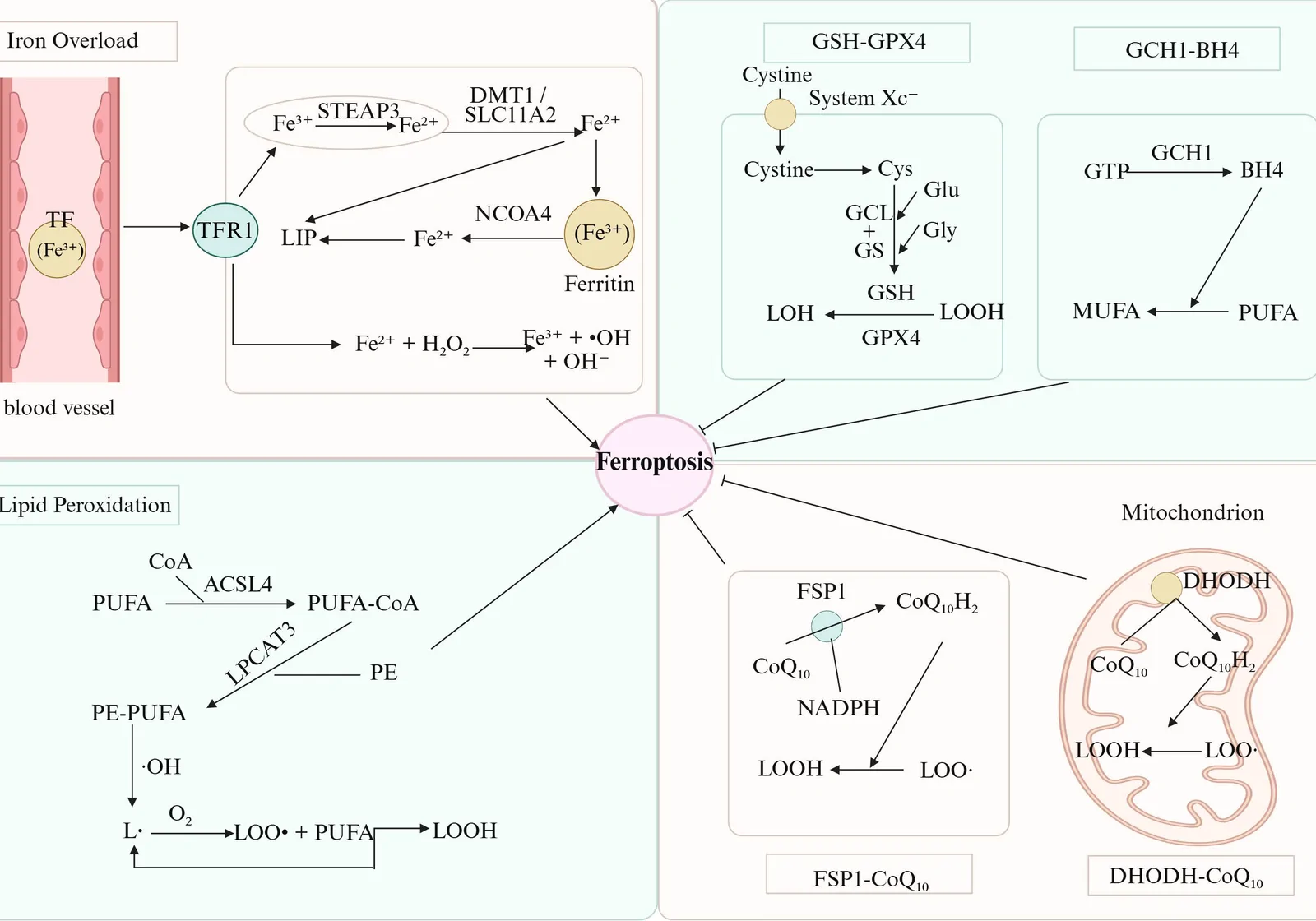
** Molecular landscape of ferroptosis drivers and defense systems.** Iron-dependent lipid peroxidation is fundamental to ferroptosis execution, wherein Fe²⁺ accumulation, tightly regulated by PTM (e.g., phosphorylation, ubiquitination) of key iron metabolic proteins, drives ROS generation via Fenton reactions. Specifically, TFR1-mediated Fe³⁺ uptake and subsequent reduction to Fe²⁺ by STEAP3 is modulated by phosphorylation of TFR1, which alters its membrane localization and iron transport activity. NCOA4-driven ferritinophagy, which releases Fe²⁺ from ferritin, is finely tuned by ubiquitination of NCOA4 and ferritin subunits, governing lysosomal degradation efficiency. Additionally, divalent metal transporter 1 (DMT1/SLC11A2)-mediated Fe²⁺ absorption is regulated by PTM to maintain labile iron pool (LIP) homeostasis. Pro-ferroptotic ACSL4 and LPCAT3 enzymes prepare the membrane for lethal oxidation by inserting PUFAs into phospholipids. Lipid hydroperoxides are reduced to non-toxic alcohols by the canonical System Xc^-^-GSH-GPX4 axis, which governs cellular survival. This protection is reinforced by the introduction of GPX4-independent routes such as the plasma membrane FSP1-CoQ10 system, the mitochondrial DHODH-CoQ10 axis and GCH1-BH4 pathway that together function as radical-trapping antioxidants preventing membrane rupture. **PTM:** Post-translational modification;** ROS:** Reactive oxygen species; **TFR1:** Transferrin Receptor 1; **STEAP3:** Six-Transmembrane Epithelial Antigen of Prostate 3;** NCOA4:** Nuclear Receptor Coactivator 4; **DMT1/SLC11A2:** Divalent metal transporter 1; **ACSL4:** Acyl-CoA synthetase long-chain family member 4; **LPCAT3:** Lysophosphatidylcholine acyltransferase 3; **LIP:** Labile iron pool; **PUFAs:** Polyunsaturated fatty acids; **System Xc^-^:** Cystine/glutamate antiporter system xc^-^;** GSH:** Glutathione; **GPX4:** Glutathione peroxidase 4; **FSP1:** ferroptosis suppressor protein 1; **CoQ10:** Coenzyme Q10; **DHODH:** Dihydroorotate dehydrogenase; **GCH1:** GTP cyclohydrolase 1; **BH4:** Tetrahydrobiopterin.

**Figure 3:**
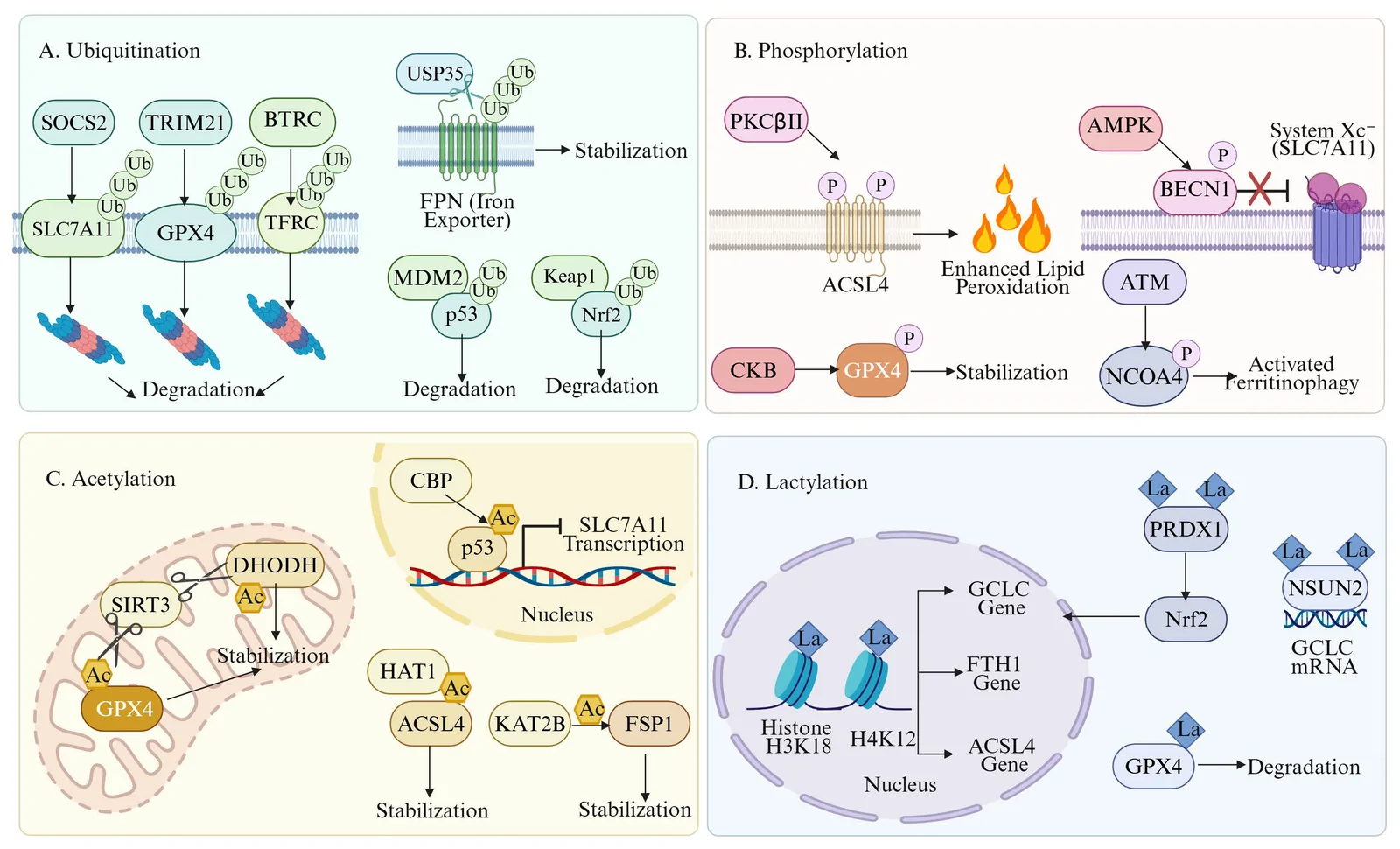
** PTM networks orchestrating ferroptosis sensitivity.** The schematic integrates four major PTM—ubiquitination, phosphorylation, acetylation, and lactylation—in governing the stability and activity of key ferroptotic regulators. A. Ubiquitination-mediated Turnover: E3 ligases (e.g., SOCS2, TRIM21) drive the proteasomal degradation of SLC7A11 and GPX4. Iron homeostasis is tuned by βTrCP-mediated TFRC degradation (reducing iron uptake) and USP35-mediated FPN stabilization (enhancing iron export). MDM2 and Keap1 control the degradation of p53 and Nrf2, respectively. B. Phosphorylation-dependent Switching: PKCβII activates ACSL4 via Thr328 phosphorylation to promote lipid peroxidation. Stress-induced AMPK phosphorylates BECN1 (S90/93/96) to block SLC7A11, while AKT-activated CKB stabilizes GPX4 via Ser104 phosphorylation. ATM-mediated NCOA4 phosphorylation at Ser550 triggers ferritinophagy and increases the labile iron pool. C. Acetylation-mediated Stability: Mitochondrial SIRT3 deacetylates and stabilizes GPX4 and DHODH to neutralize ROS. In the nucleus, CBP-mediated p53 acetylation represses SLC7A11 transcription. HAT1 and KAT2B maintain the stability of ACSL4 and FSP1 through site-specific acetylation. D. Lactylation-driven Epigenetic and Functional Remodeling: Histone lactylation (H3K18la/H4K12la) activates the transcription of GCLC, FTH1, and ACSL4. Non-histone lactylation of PRDX1 promotes Nrf2 nuclear entry, and NSUN2-La stabilizes GCLC mRNA. Conversely, direct GPX4 lactylation leads to its protein destabilization. **PTM:** Post-Translational Modification; **SOCS2:** Suppressor of Cytokine Signaling 2; **TRIM21:** Tripartite motif-containing protein 21; **SLC7A11:** Solute Carrier Family 7 Member 11; **GPX4:** Glutathione peroxidase 4; **βTrCP:** Beta-transducin repeat-containing protein; **TFRC:** Transferrin receptor protein 1; **USP35:** Ubiquitin-specific-processing protease 35;** FPN:** Ferroportin; **MDM2:** Mouse double minute 2 homolog; **Keap1**:Kelch-like ECH-associated protein 1; **p53:** Tumor protein p53; **Nrf2:** Nuclear factor erythroid 2-related factor 2; **PKCβII:** Protein kinase C beta II; **ACSL4:** Acyl-CoA synthetase long-chain family member 4; **Thr328:** Threonine 328; **AMPK:** 5'-AMP-activated protein kinase;** BECN1:** Beclin 1; **AKT:** AKT serine/threonine kinase; **CKB:** Creatine kinase B-type; **ATM:** Ataxia telangiectasia mutated; **NCOA4:** Nuclear receptor coactivator 4; **Ser550:** Serine 550; **SIRT3:** Sirtuin 3; **DHODH:** Dihydroorotate dehydrogenase; **ROS:** Reactive oxygen species; **CBP:** CREB-binding protein; **HAT1:** Histone acetyltransferase 1; **KAT2B:** Lysine acetyltransferase 2B; **FSP1:** Ferroptosis suppressor protein 1; **H3K18la:** Histone H3 lysine 18 lactylation; **H4K12la:** Histone H4 lysine 12 lactylation; **GCLC:** Glutamate-cysteine ligase catalytic subunit; **FTH1:** Ferritin heavy chain 1;** PRDX1:** Peroxiredoxin 1; **NSUN2:** NOP2/Sun domain family, member 2.

**Figure 4:**
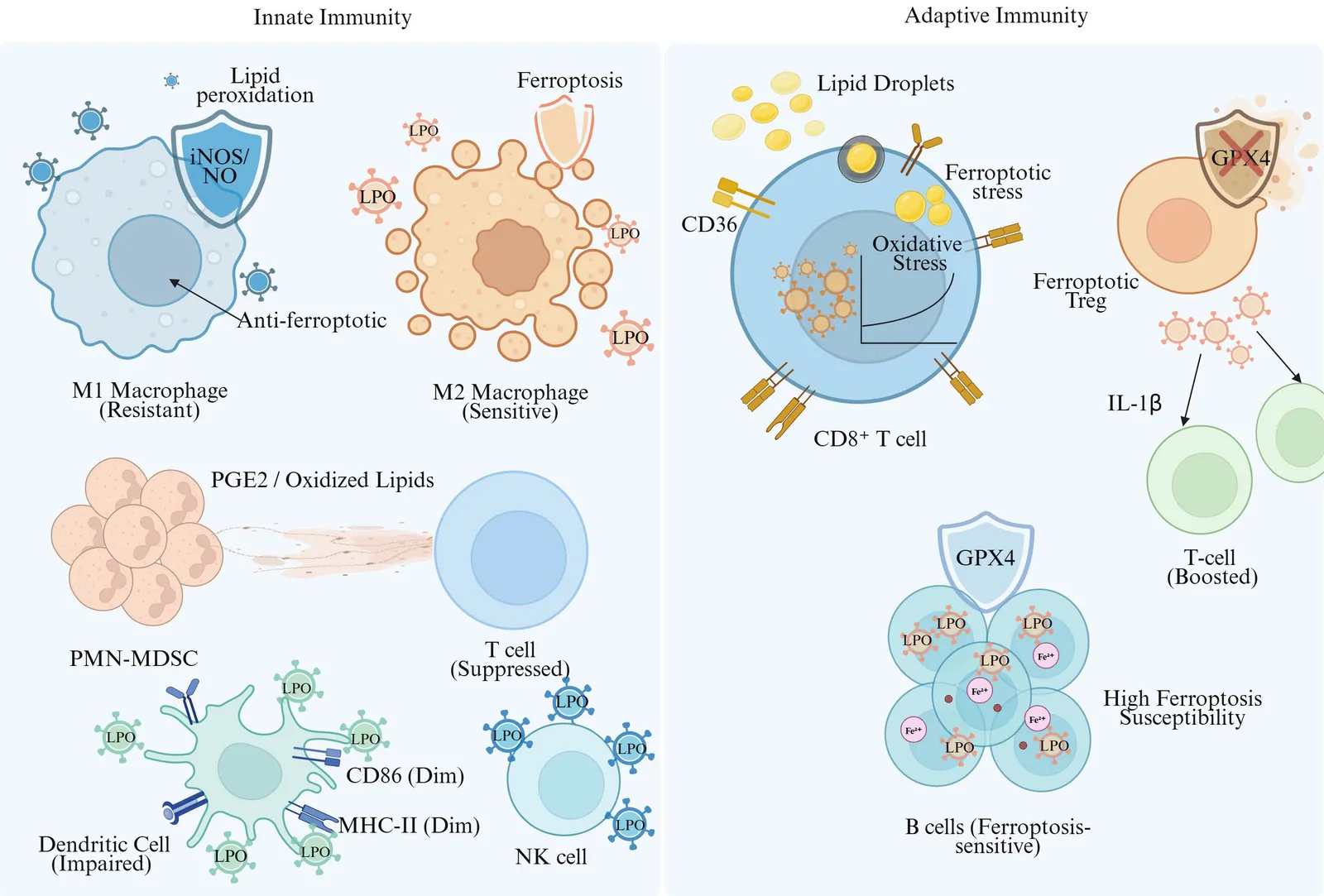
** Impact of ferroptosis on immune cell landscapes in the tumor microenvironment.** Ferroptosis acts as a multifaceted regulator of immunity through cell-type-specific vulnerabilities. In innate immunity, M1-type macrophages exhibit resistance via iNOS/NO expression, while ferroptosis induction in M2-type macrophages promotes antitumor repolarization. Conversely, spontaneous ferroptosis in PMN-MDSCs releases immunosuppressive oxygenated lipids, such as PGE_2_, while lipid peroxidation in DCs and NK cells impairs their maturation and metabolic activity. In adaptive immunity, CD36-mediated fatty acid uptake sensitizes CD8^+^ T cells to ferroptosis, attenuating their cytotoxic capacity. In contrast, GPX4 deficiency in Tregs triggers ferroptosis and the release of IL-1β, which paradoxically bolsters antitumor immunity. Additionally, B-cell subsets, specifically B1 and marginal zone B cells, exhibit a unique and high dependency on the GPX4 system for survival. **iNOS/NO:** Inducible nitric oxide synthase/Nitric oxide; **PMN-MDSCs:** Polymorphonuclear myeloid-derived suppressor cells;** PGE_2_:** Prostaglandin E_2_; **DCs:** Dendritic cells; **NK:** Natural killer; **GPX4:** Glutathione peroxidase 4; **Tregs:** Regulatory T cells; **IL-1β:** Interleukin-1β.

**Figure 5:**
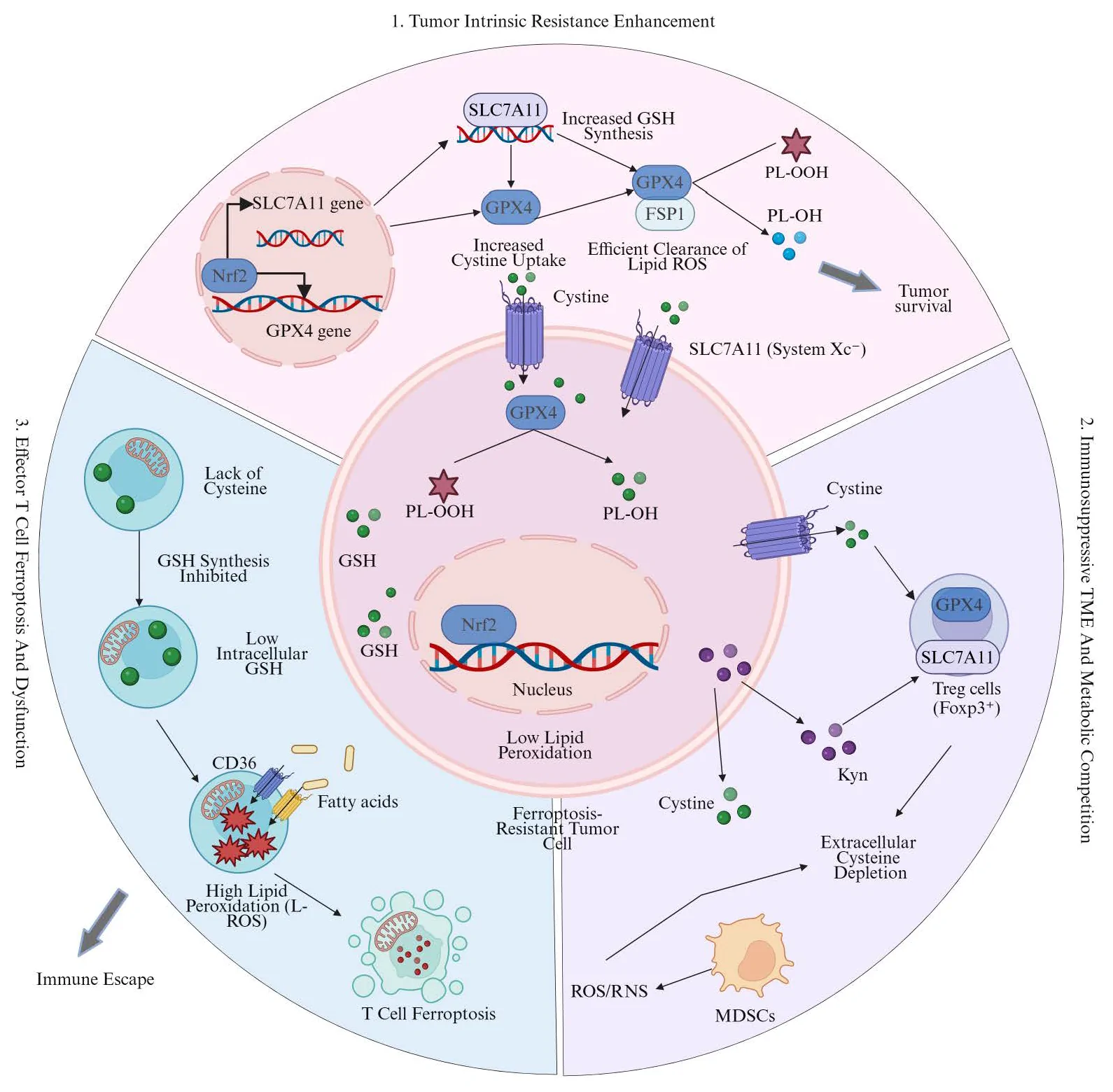
** Mechanisms of Ferroptosis-Mediated Tumor Immune Escape.** This figure illustrates the process of tumor immune escape through a radial schematic, detailing how tumor cells leverage intrinsic resistance mechanisms and an immunosuppressive microenvironment to induce effector T-cell death. By activating the transcription factor Nrf2, tumor cells regulate the expression of antioxidant genes—such as SLC7A11, GPX4, and FSP1—to establish a robust internal defense system against ferroptotic stress. Within the TME, tumor cells and Tregs, which exhibit high GPX4 expression and inherent resistance, competitively deplete extracellular cystine and cysteine. Furthermore, inhibitory cells such as MDSCs release ROS/RNS, while tumor-derived Kyn activates Tregs. These combined actions lead to the exhaustion of critical nutrients (cysteine) in the TME, fostering an inhibitory immune landscape. Effector CD8^+^ T cells are highly dependent on exogenous cysteine to maintain intracellular GSH levels. The depletion of cysteine in the microenvironment impairs GSH synthesis within T cells. Coupled with CD36-mediated fatty acid uptake, which elevates the risk of lipid peroxidation, this leads to the severe accumulation of intracellular L-ROS. Ultimately, this triggers T-cell ferroptosis and the loss of cytotoxic function, resulting in tumor immune escape. **Nrf2:** Nuclear factor erythroid 2-related factor 2; **SLC7A11:** Solute Carrier Family 7 Member 11; **GPX4:** Glutathione peroxidase 4; **FSP1:** Ferroptosis suppressor protein 1; **TME:** Tumor microenvironment; **Tregs:** Regulatory T cells; **MDSCs:** Myeloid-derived suppressor cells; **ROS/RNS:** Reactive oxygen and nitrogen species; **Kyn:** Kynurenine; **GSH:** glutathione; **L-ROS:** lipid peroxides.

**Figure 6:**
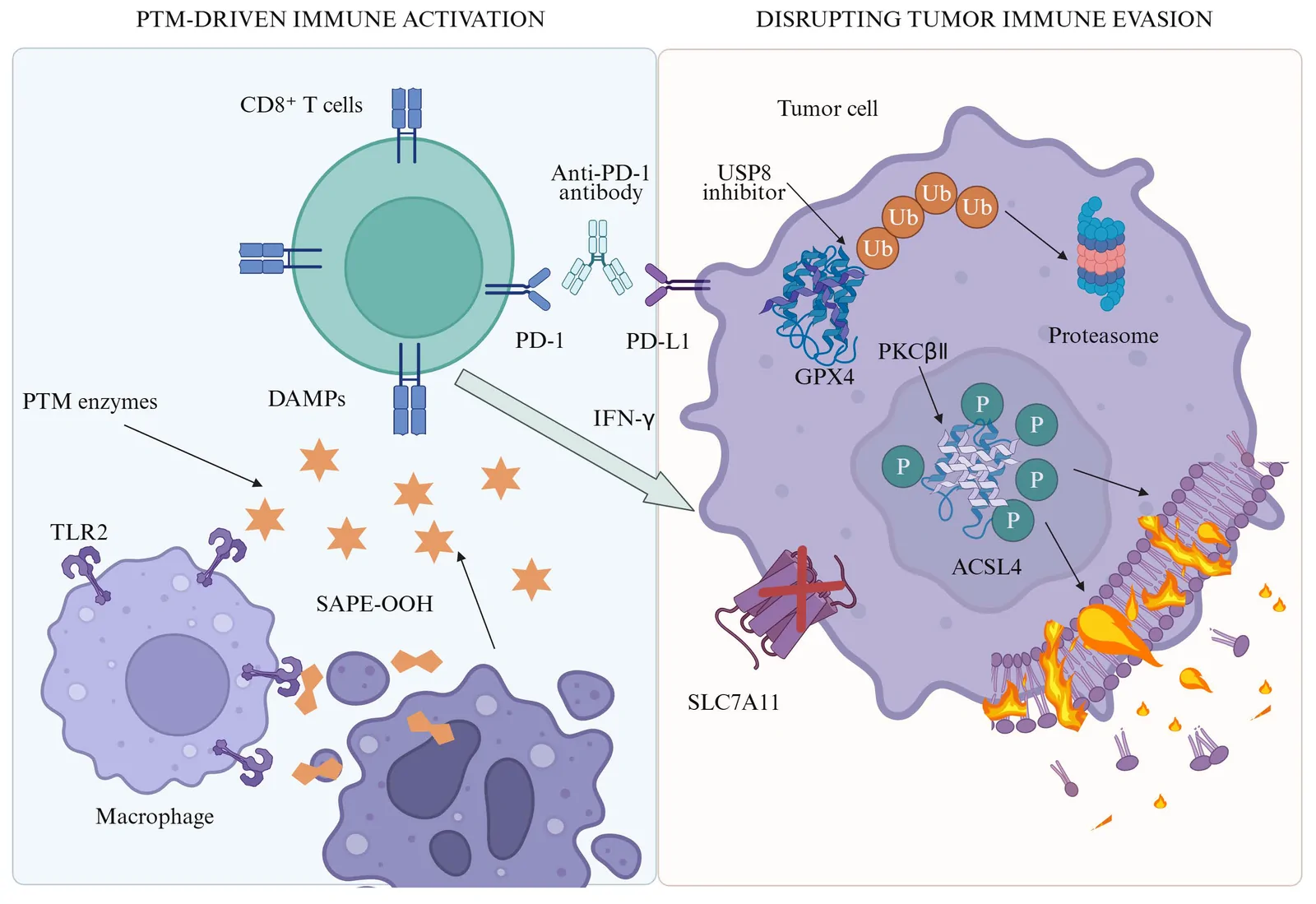
** PTM-mediated regulation of ferroptosis in immune activation and tumor eradication.** This figure illustrates the dual role of PTM in regulating ferroptosis and the interaction between tumor immunology and ferroptosis. Ferroptosis in tumor cells leads to immunogenic cell death, releasing DAMPs and expressing the SAPE-OOH signal. These signals are regulated by PTM enzymes such as OTUD1, which activates macrophages via TLR2 and promotes the infiltration and maturation of CD8+ T cells. Infiltrating T cells release IFN-γ, which inhibits the SLC7A11 transporter on tumor cells through epigenetic mechanisms, forming a positive feedback loop that enhances the efficacy of anti-PD-1/PD-L1 immunotherapy. Targeted intervention in tumor cell post-translational modification (e.g., USP8 inhibitors) can trigger GPX4 ubiquitination and degradation. Simultaneously, PKCβII-mediated ACSL4 phosphorylation enhances lipid peroxidation, making tumors more sensitive to ferroptosis. **PTM:** Post-translational modification;** DAMPs:** Damage-associated molecular patterns; **SAPE-OOH:** 1-Stearoyl-2-arachidonoyl-sn-glycero-3-phosphoethanolamine hydroperoxide; **OTUD1:** OTU domain containing 1; **TLR2:** Toll-like receptor 2; **IFN-γ:** Interferon γ; **SLC7A11:** Solute carrier family 7 member 11; **PD-1/PD-L1:** Programmed cell death protein 1/Programmed death-ligand 1; **USP8:** Ubiquitin-specific-processing protease 8; **GPX4:** Glutathione peroxidase 4; **PKCβII:** Protein kinase C beta II; **ACSL4:** Acyl-CoA synthetase long-chain family member 4.

**Figure 7:**
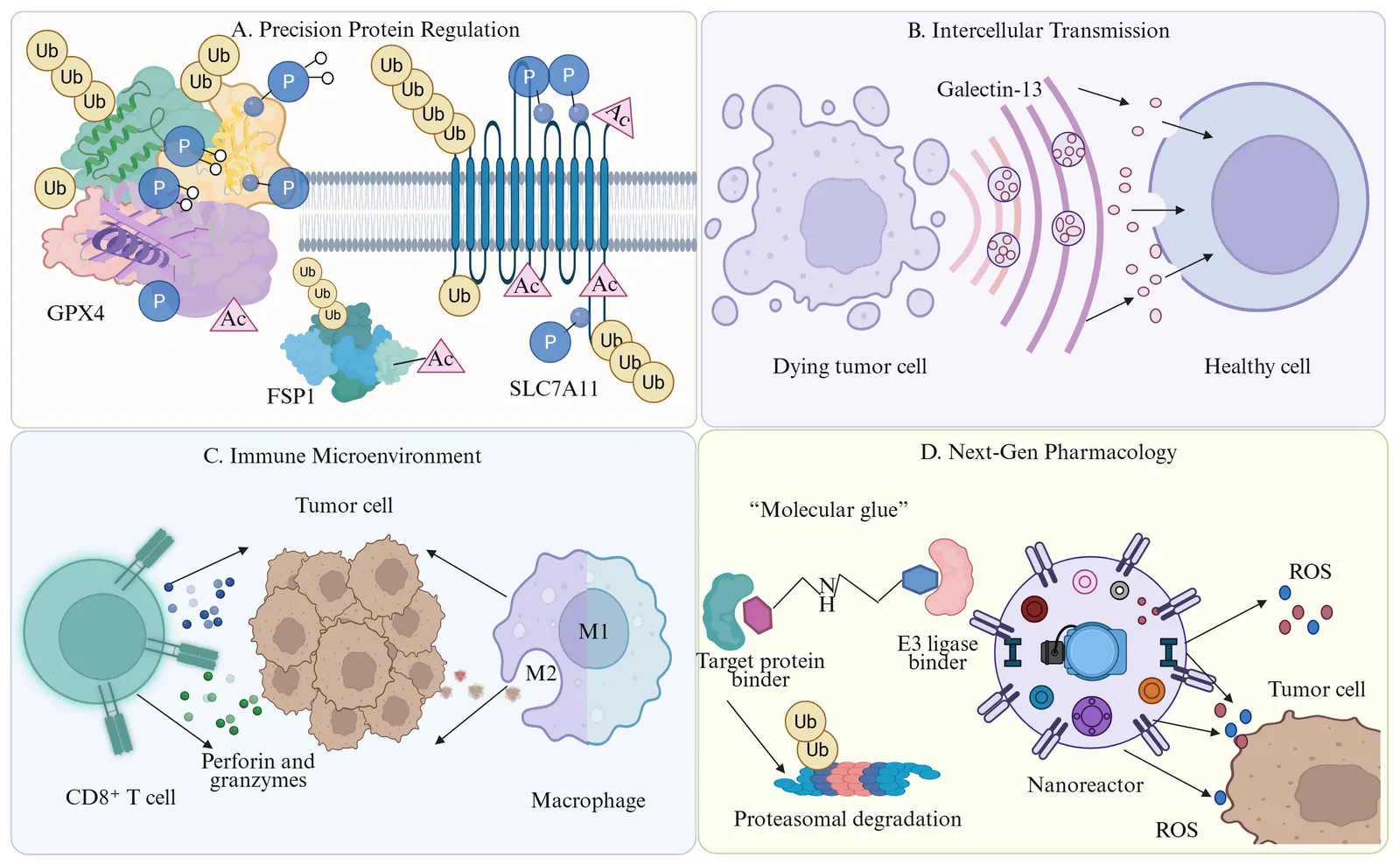
** Strategic Directions in the Regulation of Ferroptosis by PTM.** This figure highlights the four pivotal research directions at the intersection of ferroptosis and tumor therapy. (A) Precision PTM Regulation: Development focuses on the dynamic control of core proteins such as GPX4, SLC7A11, and FSP1 through varied PTM, which dictate their stability and cellular response. (B) Intercellular Ferroptosis Transmission: Emerging research identifies "wave-like" death propagation, where signals like Galectin-13 facilitate the spread of ferroptosis from dying cells to healthy neighbors, reshaping the tumor landscape. (C) Immune Microenvironment Remodeling: PTM-mediated strategies aim to differentially protect antitumor immune cells (e.g., CD8^+^ T cells) while sensitizing tumor cells, often involving the repolarization of M2 macrophages to the antitumor M1 phenotype. (D) Advanced Pharmacological Interventions: The field is moving toward high-precision tools, including PROTAC for targeted protein degradation and smart nanoreactors designed for site-specific ferroptosis induction, to maximize therapeutic efficacy and minimize off-target toxicity. **PTM:** Post-translational modification; **GPX4:** Glutathione peroxidase 4; **SLC7A11:** Solute carrier family 7 member 11; **FSP1:** Ferroptosis suppressor protein 1; **PROTAC:** Proteolysis-targeting chimera.

**Figure 8:**
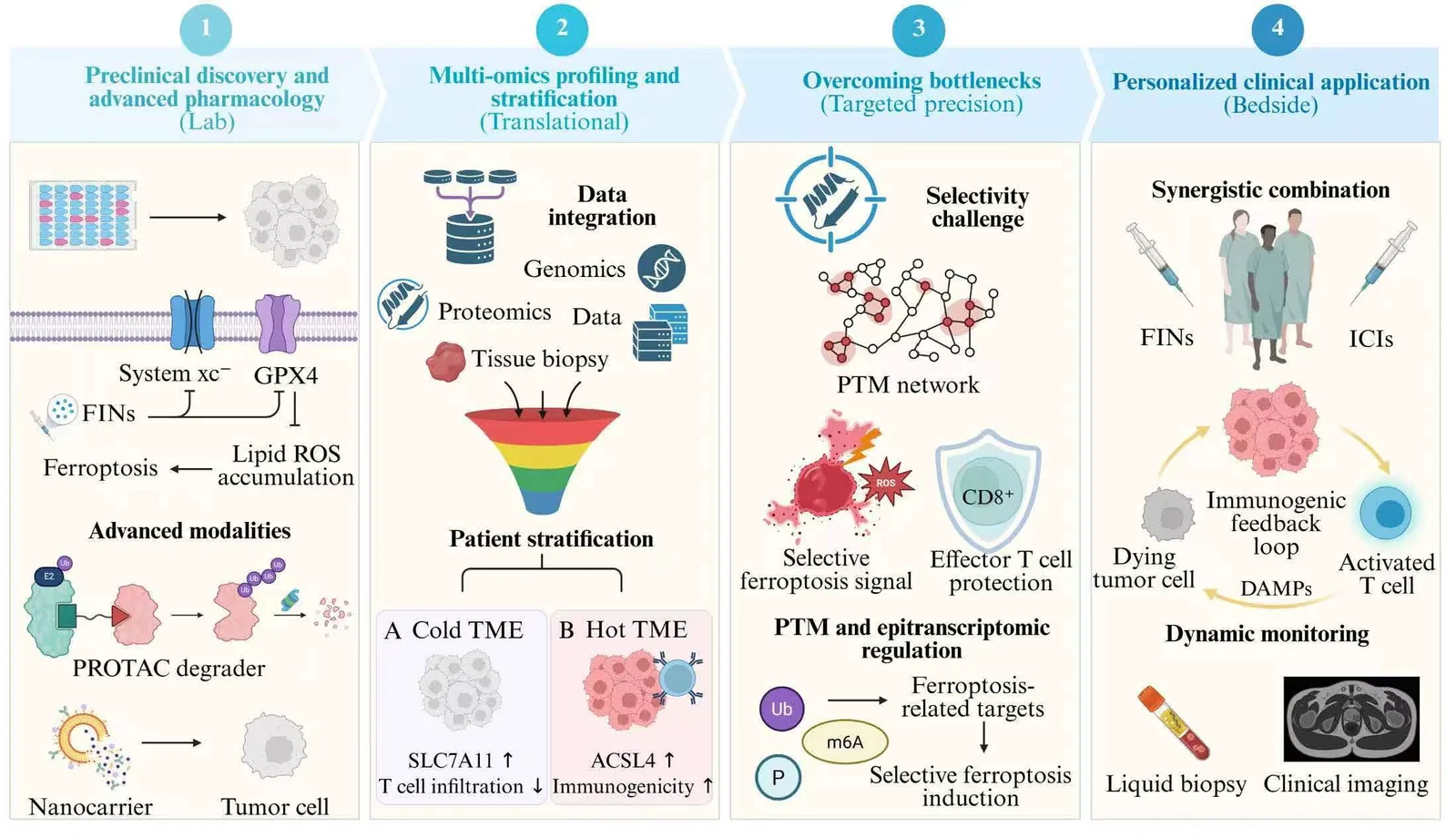
** The Clinical Translation Roadmap for Ferroptosis-Based Therapy in Cancer.** This schematic delineates the comprehensive progression of ferroptosis-targeted strategies from preclinical development to personalized clinical application across four interconnected phases. (1) Preclinical Discovery and Advanced Pharmacology: In the laboratory setting, core ferroptosis dependencies (e.g., System xc⁻ and GPX4) are targeted utilizing standard FINs alongside next-generation pharmacological modalities, including PROTAC degraders and smart nanocarriers, to enhance tumor-specific payload delivery. (2) Multi-Omics Profiling and Stratification: Translational research integrates multi-omics data (genomics and proteomics) derived from tissue biopsies to achieve patient stratification. Based on biomarker signatures, the TME is classified into "Cold" or "Hot" phenotypes to guide therapeutic interventions. (3) Overcoming Bottlenecks (Targeted Precision): A major clinical challenge is selectively inducing ferroptotic signaling (e.g., ROS accumulation) in malignant cells while preserving the structural and functional integrity of effector CD8⁺ T cells. Concurrently, navigating the highly redundant and complex post-translational modification (PTM) networks (e.g., ubiquitination [Ub], phosphorylation [P], and m⁶A RNA methylation) is essential to overcome compensatory resistance mechanisms. (4) Personalized Clinical Application: At the bedside, the synergistic co-administration of FINs and ICIs establishes a robust immunogenic positive feedback loop, wherein dying tumor cells release DAMPs to continuously prime and activate T cells. Finally, longitudinal dynamic monitoring via liquid biopsies and clinical imaging facilitates the real-time evaluation and optimization of personalized treatment regimens. **FINs:** ferroptosis inducers; **PROTAC:** proteolysis-targeting chimera; **TME:** tumor microenvironment; **PTM:** post-translational modification; **ROS:** reactive oxygen species; **ICIs:** immune checkpoint inhibitors; **DAMPs:** damage-associated molecular patterns.

**Table 1:** PTM regulatory atlas of ferroptosis-related proteins

Regulatory Pathway	Target Protein	Type of PTM	Key Modification Sites/Enzymes	Effect on Protein Function	Effect on Ferroptosis	Representative Upstream Signals	Ref.
SLC7A11-GSH-GPX4 axis	SLC7A11	Ubiquitination	WWP1	WWP1 induces SLC7A11 degradation.	Promotion of ferroptosis	QKI-5	332
SLC7A11-GSH-GPX4 axis	GPX4	Ubiquitination	KLHL8	GPX4 undergoes autophagic lysosomal degradation via TAX1BP1.	Promotion of ferroptosis	OGD/R	333
SLC7A11-GSH-GPX4 axis	GPX4	Ubiquitination	HSP90	Promotes GPX4 ubiquitination and subsequent proteasomal degradation.	Promotion of ferroptosis	ADG	334
SLC7A11-GSH-GPX4 axis	GPX4	Ubiquitination	STUB1	K48-linked polyubiquitination that mediates GPX4 leads to its degradation.	Promotion of ferroptosis	ORI exposure	335
SLC7A11-GSH-GPX4 axis	SLC7A11	Ubiquitination	TNFAIP3	It mediates ubiquitination modification of SLC7A11, leading to protein degradation.	Promotion of ferroptosis	GT	336
SLC7A11-GSH-GPX4 axis	SLC7A11	Deubiquitination	OTUB1	The ubiquitin chain on SLC7A11 was removed to enhance its protein stability.	Inhibition of ferroptosis	\	337
SLC7A11-GSH-GPX4 axis	GPX4	Deubiquitination	USP5	USP5 deubiquitinates and stabilizes GPX4.	Inhibition of ferroptosis	FASN	15
SLC7A11-GSH-GPX4 axis	SLC7A11	Deubiquitination	OTUB1	The ubiquitin chain on SLC7A11 was removed to prevent its degradation by the proteasome.	Inhibition of ferroptosis	dRib	338
SLC7A11-GSH-GPX4 axis	SLC7A11	Ubiquitination	SOCS6	It mediates ubiquitination of SLC7A11 and induces its degradation by the proteasome.	Promotion of ferroptosis	\	339
SLC7A11-GSH-GPX4 axis	GPX4	Deubiquitination	OTUD4	Deubiquitylation of GPX4 prevents its degradation by proteasomes and autophagy.	Inhibition of ferroptosis	RHEB	340
SLC7A11-GSH-GPX4 axis	GPX4	Phosphorylation	CKB/S104	Blocking the binding of its molecular partner HSC70, inhibiting chaperon-mediated autophagy (CMA) degradation, and stabilizing GPX4 protein level.	Inhibition of ferroptosis	IGF1R/AKT axis	100
SLC7A11-GSH-GPX4 axis	GPX4	Phosphorylation	STK38/S45	With the assistance of SCRN1, GPX4 is phosphorylated to inhibit HSC8-mediated CMA degradation.	Inhibition of ferroptosis	SCRN1 overexpression	341
SLC7A11-GSH-GPX4 axis	SLC7A11	Phosphorylation	mTORC2/S26	Direct inhibition of SLC7A11 transport activity reduces cysteine uptake	Promotion of ferroptosis	EGFRvIII/Growth factor signaling	342
SLC7A11-GSH-GPX4 axis	GPX4	Hydroxylation	PHD1/P70	PHD1-mediated p70 hydroxylation prevents GPX4 degradation, thereby stabilizing GPX4.	Inhibition of ferroptosis	PSAT1 upregulation	151
SLC7A11-GSH-GPX4 axis	GPX4	Acetylation	\	Inhibits GPX4 function and disrupts mitochondrial redox homeostasis.	Promotion of ferroptosis	Cadmium exposure and SIRT3 inhibition	115
SLC7A11-GSH-GPX4 axis	GPX4	Lactylation	EGFR/SRC	Enhances GPX4 function and suppresses oxidative stress.	Inhibition of ferroptosis	High-glucose environment	343
SLC7A11-GSH-GPX4 axis	GPX4	Lactylation	KAT5/KAT8	Lactylation of GPX4 reduces its enzymatic antioxidant activity.	Promotion of ferroptosis	High-glucose environment	344
SLC7A11-GSH-GPX4 axis/Transcriptional regulation	Histone H4/GCLC	Histone lactylation	p300, HDAC1; H4K12la	H4K12la upregulates GCLC expression and increases GSH synthesis.	Inhibition of ferroptosis	Lactate	132
FSP1-CoQ10 axis	FSP1	Acetylation	KAT2B/K168	Inhibits K29-linked polyubiquitination and prevents proteasomal degradation.	Inhibition of ferroptosis	Acetyl-CoA	119
FSP1-CoQ10 axis	FSP1	Deubiquitination	USP29	USP29 deubiquitinates and stabilizes FSP1.	Inhibition of ferroptosis	\	345
FSP1-CoQ10 axis	FSP1	Deubiquitination and Myristoylation	Src kinase	Src promotes deubiquitination and enhances myristoylation of FSP1.	Inhibition of ferroptosis	MUC1	346
FSP1-CoQ10 axis	FSP1	Myristoylation	NMT2	Facilitates membrane recruitment of FSP1 and its CoQ10-reducing activity.	Inhibition of ferroptosis	NADPH	347
FSP1-CoQ10 axis	FSP1	N4-acetylcytidine	NAT10	Promotes FSP1 protein stability and prevents its degradation.	Inhibition of ferroptosis	LPS	348
DHODH-CoQ10 axis	DHODH	Acetylation	\	Impairs DHODH activity and leads to decreased CoQ10H2 levels.	Promotion of ferroptosis	Cisplatin induction and SIRT3 inhibition	349
Lipid metabolism	ACSL4	Ubiquitination	NEDD4L	Mediates ubiquitination of ACSL4 and induces its proteasomal degradation.	Inhibition of ferroptosis	Paeonol	350
Lipid metabolism	ACSL4	Ubiquitination	RNF5	Targets ACSL4 for ubiquitination and proteasomal degradation.	Inhibition of ferroptosis	Ischemia-reperfusion stress	351
Lipid metabolism	ACSL4	DeSUMOylation	SENP1	Circ_0002638 enhances SENP1-mediated deSUMOylation of ACSL4, leading to inhibition of ACSL4 function.	Inhibition of ferroptosis	Circ_0002638	352
Lipid metabolism	ACSL4	Phosphorylation	PKCβII	Activates ACSL4 enzymatic activity and promotes biosynthesis of PUFAs-containing phospholipids.	Promotion of ferroptosis	Lipid peroxides	102
Lipid metabolism	ACSL4	Phosphorylation and Ubiquitination	CDK1/S447; UBR5	CDK1-mediated phosphorylation recruits UBR5 to mediate ACSL4 ubiquitination and proteasomal degradation.	Inhibition of ferroptosis	Loss of m⁶A modification	353
Lipid metabolism	ACSL4	Acetylation	HAT1/K383	Blocks FBXO10-mediated K48-linked ubiquitination and enhances protein stability.	Promotion of ferroptosis	HDAC2/SIRT3 axis	118
Lipid metabolism	ACSL4	Acetylation	KAT2B/K500, K571, K692	Increases affinity for HSPA8, thereby driving CMA-mediated degradation.	Inhibition of ferroptosis	\	354
Lipid metabolism	ACSL4	Deubiquitination	USP22	Removes ubiquitin chains and markedly improves ACSL4 protein stability.	Promotion of ferroptosis	High-glucose environment	355
Lipid metabolism	ACSL4	Lactylation	SIRT3/K412	Direct lactylation of ACSL4 and H3K18 increases their expression and activity.	Promotion of ferroptosis	Glycolysis	356
Lipid metabolism/Transcriptional regulation	Histone H3/METTL3	Histone lactylation	p300; H3K18la	H3K18la at the METTL3 promoter enhances its expression and stabilizes ACSL4 mRNA.	Promotion of ferroptosis	Glycolysis	129
Iron metabolism	NCOA4	Deubiquitination	YOD1	Prevents degradation of NCOA4 and maintains its protein stability.	Promotion of ferroptosis	LPS	357
Iron metabolism	TFRC	O-GlcNAcylation	OGT	Improves TFRC protein stability and prevents its degradation.	Increased sensitivity to ferroptosis	Hexosamine biosynthetic pathway	149
Iron metabolism	NCOA4	Phosphorylation	ATM	Enhances the interaction between NCOA4 and FTH1 to drive ferritinophagy.	Promotion of ferroptosis	DSBs	104
Iron metabolism/Transcriptional regulation	LSD1/TFRC	Lactylation	\	Lactate-induced lactylation of LSD1 cooperates with FosL1 to direct transcription and repress TFRC.	Inhibition of ferroptosis	Glycolysis	358

**Table 2:** PTM-mediated crosstalk between ferroptosis and the tumor immune microenvironment

Direction of immune crosstalk	Key PTM nodes	Mediating factors	Involved immune cells	Effect of PTM on signal transduction	Final immune outcome	Ref.
Reversal of immunosuppression	METTL3ubiquitination	TRIM21	CD8⁺ T Cells	METTL3 ubiquitination leads to its proteasomal degradation, impairs SLC7A11 mRNA stability, and promotes ferroptosis.	Elevated PD-L1 expression in ferroptotic tumor cells; Increased infiltration and cytotoxicity of CD8⁺ T cells.	252
Reversal of immunosuppression	YAP deubiquitination	USP52	CD8⁺ T Cells	Deubiquitination of YAP stabilizes this protein and inhibits ferroptosis.	Enhanced infiltration and cytotoxic activity of CD8⁺ T cells; Improved responsiveness of tumor cells to PD-L1 immunotherapy.	268
Reversal of immunosuppression	RORC acetylation, SLC39A14/8, STEAP3 ubiquitination	p300, NEDD4L	NK Cells, TAMs	Acetylation of RORC drives NEDD4L transcription, thereby promoting ubiquitination and degradation of ferrotransporters and suppressing ferroptosis in NK cells.	Inhibition of ferroptosis in NK cells preserves their cytotoxic, adaptive and heat-shock functional phenotypes.	267
Immunosuppression	FHL2deubiquitination	UCHL1	CD8⁺ T Cells	Deubiquitination of FHL2 stabilizes FHL2 and inhibits the ferroptosis pathway.	Decreased activity of CD8⁺ T cells; Promotion of tumor immune escape.	359
Macrophage polarization (M2)	SELENBP1 deubiquitination	USP15	Macrophages (M2), T Cells	Deubiquitination of SELENBP1 stabilizes GPX4 and reduces lipid ROS, thereby inhibiting ferroptosis.	Promotion of M2 macrophage polarization; Reduced T-cell cytotoxicity; Altered chemokine profile.	360
Macrophage polarization (M2)	Inhibition of SLC25A10 deubiquitination	TRIM21	Macrophages (M2)	INHBA blocks ubiquitination and degradation of SLC25A10, activates the succinate/SUCNR1 axis in macrophages and the mtGSH/GPX4 axis, thus inhibiting ferroptosis.	Promotion of macrophage polarization toward an M2 phenotype; Inhibition of the phagocytic capacity of macrophages.	361
Macrophage polarization (M1)	O-/N-glycosylation	ST3GAL1; GYG2	Macrophages (M2)	Modulates glycosylation of membrane proteins and metabolic enzymes, and activates TNF and NF-κB signaling.	Polarization of TAMs toward an M1 phenotype enhances anti-tumor immunity.	312
Macrophage polarization (M2) and Metabolic competition	Inhibition of ENO1 ubiquitination	RARS1	Macrophages (M2)	RARS1 inhibits ubiquitination and degradation of ENO1, activates the PI3K/AKT pathway, upregulates GPX4, and effectively suppresses ferroptosis.	Promotion of macrophage polarization toward an M2 phenotype; Inhibition of pro-inflammatory M1 markers.	362
Macrophage polarization (M1) and Antigen presentation	PIR UFMylation inhibition	MCP, UFM1	Macrophages (M1)	Inhibition of MCP reduces UFMylation of PIR, resulting in PIR degradation, decreased GPX4 transcription, and cytoplasmic accumulation of HMGB1.	Promotion of macrophage polarization toward an M1 phenotype.	179
Antigen presentation	IREB2 deubiquitination	OTUD1	CD8⁺ T Cells	Deubiquitination of IREB2 stabilizes TFRC mRNA, increases iron uptake, and promotes ferroptosis.	Ferroptotic tumor cells release DAMPs, which recruit and activate CD8⁺ T cells.	94
Metabolic competition	RORC acetylation , Ferrotransporter deubiquitination	Acetyl-CoA, NEDD4L	NK Cells	Acetylated RORC upregulates NEDD4L, which in turn mediates ubiquitination and degradation of ferrotransporters, thereby inhibiting ferroptosis in NK cells.	Promotion of NK cell differentiation into cytotoxic and adaptive phenotypes enhances the killing effect of NK cells on HCC cells.	267
Metabolic competition	DRP1 phosphorylation	PKA	MDSCs, T Cells	Phosphorylation of DRP1 enhances endoplasmic reticulum-mitochondria contact, induces ER stress and ROS production, and triggers ferroptosis in MDSCs.	Induction of ferroptosis in MDSCs; Inhibition of CD8⁺ T-cell proliferation and effector functions.	363
Immune evasion	UPF1 ubiquitination	TRIM34	CD8⁺ T Cells, MDSCs, CAFs, Tregs, Macrophages (M2)	Ubiquitination of UPF1 stabilizes GPX4 mRNA, thereby inhibiting ferroptosis.	T-cell exclusion; Increased infiltration of MDSCs, CAFs and M2 macrophages; Reduced sensitivity to PD-1 inhibitors.	364
Metabolic competition and Immune evasion	DNA methylation	DNMTs	Macrophages (M2), Tregs	Mediates hypomethylation of the SLC7A11 promoter and activates the NRF2-GSH-GPX4 axis.	Induction of ferroptosis resistance upregulates PD-L1 expression and mediates immune escape.	365
Immune cell infiltration and checkpoint regulation	Arginine methylation	PRMT5	NK Cells, CD4⁺ T Cells	Modulates the methylation status of IL-17 and p53-related proteins, leading to enrichment of tumor-associated pathways.	High expression of PRMT5 is associated with poor prognosis and shows a significant correlation with immune cell infiltration.	366

**Table 3:** Clinical translation strategies and challenges targeting PTM in ferroptosis

Therapeutic Strategy	Representative Drugs/Techniques	Targeted PTM Sites	Combination Therapeutic Regimens	Potential Biomarkers	Major Translational Barriers/Challenges	Ref.
Epigenetic regulation	SOCS2	SLC7A11 K48-linked polyubiquitination	SOCS2 + radiotherapy	Low SOCS2 expression	Complex crosstalk within the SOCS family; Delivery of E3 ligase modulators to tumor sites.	79
Epigenetic regulation	PARP inhibitors	GPX4 K6-linked polyubiquitination	PARPi + ferroptosis inducer	BRCA1; GPX4	Complexity of the HR-deficient pathway; Potential toxicity of the PARPi-ferroptosis inducer combination.	367
Epigenetic regulation	UBR5 inhibition/small-molecule inhibitors	Smad3 K11-linked polyubiquitination	UBR5 inhibitor + 5-FU/oxaliplatin	UBR5; Smad3; K11-linked ubiquitin levels	High complexity of non-canonical (K11) ubiquitin coding; Lack of clinical-grade selective UBR5 inhibitors.	368
Epigenetic regulation	METTL3 inhibitors; m⁶A inhibitors	SLC7A11 Ubiquitination	METTL3 inhibitor + radiotherapy	METTL3; IGF2BP2; SOCS2	Dynamic nature of m⁶A modification; Off-target effects of global methyltransferase inhibition.	369
Epigenetic regulation	OTUD5 inhibitors/DUBs inhibitors	SLC7A11 Deubiquitination	OTUD5 inhibitor + paclitaxel	OTUD5	Development of highly selective small-molecule inhibitors targeting the deubiquitinase OTUD5.	370
Epigenetic regulation	iFSP1; USP29 inhibitors	FSP1 Ubiquitination/Deubiquitination	iFSP1 + conventional chemotherapeutic agents	USP29/SMURF1 expression ratio; FSP1 protein levels	Precise targeting of DUB/E3 interactions; Compensatory activation of other ferroptosis-suppressive pathways.	345
Epigenetic regulation	Toosendanin	PLK1 Deubiquitination	Toosendanin + immunotherapy	USP39 and PLK1 levels; M1/M2 macrophage ratio	Balancing the potent anti-tumor activity of toosendanin with potential systemic toxicity.	371
Epigenetic regulation	Linc01833 antagonist	SLC7A11 Ubiquitination	Linc01833 inhibitor + gemcitabine	Linc01833; WWP1	Improving the in vivo stability of antisense oligonucleotides targeting Linc01833.	332
Metabolic intervention/Epigenetic regulation	GSK3326595; FIDAS-5; methionine restriction	GPX4 (R152) symmetric dimethylation	GSK3326595 + ferroptosis inducer	PRMT5; R152-me2s	Systemic toxicity induced by methionine deprivation; Off-target effects and selectivity of systemic PRMT inhibitors.	144
Metabolic intervention/Epigenetic regulation	Tanshinone IIA; ORY-1001	SLC7A11 (K500) SUMOylation	Tanshinone IIA + conventional chemotherapy	KDM1A; PIAS4; SLC7A11	Low bioavailability of natural products such as tanshinone IIA; Precise targeting of specific SUMOylation sites.	372
Metabolic intervention/Epigenetic regulation	Enzalutamide; Erastin	SLC7A11 Ubiquitination	Enzalutamide + ferroptosis inducer	AR; NEDD4L; SLC7A11	Balancing androgen receptor antagonism and systemic ferroptosis-related toxicity in prostate cancer.	373
Metabolic intervention/Epigenetic regulation	DTX2 inhibitors; E3 ligase modulators	NCOA4 Ubiquitination	DTX2 inhibitor + iron overload agent	DTX2	Development of highly selective small-molecule inhibitors of DTX2; Delivery to the lung tumor microenvironment.	374
Metabolic intervention/Epigenetic regulation	RRM2 inhibitors	GPX4 Ubiquitination	RRM2 inhibitor + cisplatin	RRM2; GPX4	Development of RRM2 inhibitors with high specificity and low hematologic toxicity.	375
Metabolic intervention/Epigenetic regulation	Sodium butyrate	GPX4 Ubiquitination	Sodium butyrate + anti-PD-1 antibody	USP5; Butyrate	Dose standardization of short-chain fatty acids; Variability in patient responses based on microbiota composition.	376
Metabolic intervention/Epigenetic regulation	circ_0002638 silencing; SENP1 inhibitors	ACSL4 deSUMOylation	circ_0002638 knockdown + chemotherapy	circ_0002638; SENP1; SUMOylation of ACSL4	Identification of safe and efficient delivery systems for circRNA-targeted therapy.	352
Metabolic intervention/Natural products	Erianin	GPX4 Ubiquitination	Erianin + conventional chemotherapy	ROS levels; GPX4	Standardization of natural product extracts; Identification of the specific E3 ligase recruited by erianin.	317
Metabolic intervention/Natural products	DMOCPTL	GPX4 Ubiquitination	DMOCPTL + immune checkpoint inhibitors	GPX4; EGR1	Improving the systemic bioavailability and metabolic stability of parthenolide derivatives.	377
Metabolic intervention/Natural products	Nobiletin	GPX4 Ubiquitination	Nobiletin + standard chemotherapy	AKR1C1; GPX4	Identification of the specific E3 ligase recruited by the nobiletin-AKR1C1 complex.	378
Metabolic intervention/Autophagy regulation	Ginkgetin	GPX4 Ubiquitination	Ginkgetin + EGFR inhibitor	TFEB nuclear translocation; TRIM25 levels	Elucidating the crosstalk between proteasomal and lysosomal degradation pathways of GPX4.	379
Metabolic intervention/Natural products	Gingerone A	SLC7A11 Ubiquitination	Gingerone A + 5-FU/oxaliplatin	SLC7A11	Identification of the definitive E3 ligase involved in GA-induced SLC7A11 degradation.	380
Metabolic intervention/Natural products	Taraxasterol	GPX4 Ubiquitination	Taraxacum + radiotherapy	Nrf2 transcriptional activity; MIB2 levels	Determination of the exact binding site of taraxasterol on Nrf2; Low in vivo bioavailability of natural triterpenoids.	381
Metabolic intervention/Natural products	Berberine	GPX4 Ubiquitination	Berberine + cisplatin	USP51; GPX4	Elucidating the precise regulatory role of USP51 in GPX4 ubiquitination and deubiquitination.	382
Metabolic intervention/Natural products	Resveratrol	GPX4 Ubiquitination	Resveratrol + conventional chemotherapy	NEDD4L; GPX4	Rapid metabolism and low systemic bioavailability of resveratrol in vivo.	383
Metabolic intervention/Natural products	Bufotalin	GPX4 Ubiquitination	Bufotalin + ferroptosis inducer	GPX4; ROS levels	Controlling the systemic toxicity associated with cardenolides such as bufotalin.	384
Metabolic intervention/Natural therapy	Xihuang Pill	SLC7A11 Deubiquitination	Xihuang Pill + ICIs	OTUB1; SLC7A11; lipid peroxidation markers	Identification of specific active components in the complex Xihuang Pill formulation; Clinical standardization.	385
Metabolic intervention	Andrographolide	GPX4 Ubiquitination	Andrographolide + immunotherapy	HSP90/GPX4 interaction level; mitochondrial dysfunction markers	Characterization of the precise molecular docking of andrographolide on the HSP90-GPX4 interaction interface.	334
Kinase inhibitors	Imatinib	GPX4 (K191) Ubiquitination and degradation	Imatinib + RSL3	STUB1; GPX4	Primary and acquired resistance to imatinib; 2. Off-target effects of systemic GPX4 inhibition.	386
Kinase inhibitors	Sorafenib	FSP1 Ubiquitination	Sorafenib + ferroptosis inducer	TRIM54; FSP1	Heterogeneity of hepatocellular carcinoma; 2. Metabolic adaptation of tumor cells to bypass FSP1-dependent protection.	91
Kinase inhibitors	MI-2	GPX4 Ubiquitination	MALT1 inhibitor + sorafenib/regorafenib	MALT1; RC3H1; GPX4	Systemic MALT1 inhibition may impair T-cell function; 2. Balancing anti-tumor efficacy and immune homeostasis.	387
Kinase inhibitors/Epigenetic regulation	Dasatinib; anti-MUC1 antibody	FSP1 Deubiquitination and Myristoylation	Dasatinib + ferroptosis inducer	MUC1	Complexity of dual PTM regulation; 2. Potential systemic toxicity of multi-target kinase inhibitors.	346
Targeted protein degradation	GPX4-AUTAC	GPX4 Ubiquitination	GPX4-AUTAC + ICIs	GPX4; TRAF6; p62	Optimization of the pharmacokinetic properties of macromolecular chimeras; 2. Ensuring long-term biosafety and avoiding off-target autophagic stress.	388
DUB-targeted therapy/Epigenetic regulation	TCF12 activators; OTUB1 inhibitors	SLC7A11 Deubiquitination	TCF12 overexpression + cisplatin	TCF12; OTUB1; SLC7A11	Pharmacological targeting of the TCF12 transcription factor remains challenging; Specificity of OTUB1 inhibitors.	389
DUB inhibitors/radiotherapy	USP14 inhibitors	GPX4 (K48) Deubiquitination	USP14 inhibitor + radiotherapy	TRIM14/USP14 complex; GPX4	Development of USP14-specific inhibitors with optimal pharmacokinetic profiles.	390

## Data Availability

Data sharing not applicable to this article as no datasets were generated or analyzed during the current study. All information is derived from publicly available articles and datasets.

## Abbreviations

ROS: Reactive oxygen species
PTM: Post-Translational Modification
CTLs: Cytotoxic T Lymphocytes
IFN-γ: Interferon γ
SLC7A11: Solute Carrier Family 7 Member 11
DAMPs: Damage-Associated Molecular Patterns
DCs: Dendritic Cells
FASN: Fatty Acid Synthase
USP5: Ubiquitin-specific protease 5
GPX4: Glutathione Peroxidase 4
ACSL4: Acyl-CoA synthetase long-chain family member 4
GSH: Glutathione
System xc⁻: Cystine/glutamate antiporter system
CoQ10: Coenzyme Q10
NRF2: Nuclear factor erythroid 2-related factor 2
RSL3: RAS-selective lethal 3
FSP1: Ferroptosis suppressor protein 1
NAD(P)H: Nicotinamide adenine dinucleotide (phosphate) hydride
GCH1: GTP cyclohydrolase 1
BH4: Tetrahydrobiopterin
LPCAT3: Lysophosphatidylcholine acyltransferase 3
PUFAs: Polyunsaturated Fatty Acids
ALOX15: Arachidonate 15-Lipoxygenase-1
LIP: Labile Iron Pool
TF: Transferrin
TFR1: Transferrin Receptor 1
STEAP3: Six-Transmembrane Epithelial Antigen of Prostate 3
DMT1: Divalent Metal Transporter 1
NCOA4: Nuclear Receptor Coactivator 4
FPN: Ferroportin
IRP: Iron Regulatory Protein
IRE: Iron Responsive Element
BAP1: BRCA1-associated protein 1
KRAS: Kirsten rat sarcoma viral oncogene homolog
ICIs: Immune checkpoint inhibitors
IKE: Imidazole ketone erastin
DUBs: Deubiquitinating enzymes
TRIM26: Tripartite motif-containing protein 26
SOCS2: Suppressor of cytokine signaling 2
OTUB1: OTU deubiquitinase 1
βTrCP: β-transducin repeat-containing protein
IREB2: Iron Responsive Element Binding Protein 2
SCD: Stearoyl-CoA desaturase
Nedd4L: Neural precursor cell expressed developmentally downregulated protein 4-like
ATP: Adenosine Triphosphate
AKT/PKB: Protein kinase B
CKB: Creatine Kinase B
HSC70: Heat shock cognate 71 kDa protein
AMPK: AMP-activated Protein Kinase
BECN1: Beclin 1
PKCβII: protein kinase C beta II
HSPB1: Heat Shock Protein Beta 1
ATM: Ataxia Telangiectasia Mutated
TFRC: Transferrin Receptor
YAP/TAZ: Yes-associated protein/Transcriptional co-activator with PDZ-binding motif
SIRT3: sirtuin 3
HAT1: Histone acetyltransferase 1
FBXO10: F-box protein 10
MARCHF6: Membrane associated ring-CH-type finger 6
KAT5: Lysine acetyltransferase 5
m⁶A: N6-methyladenosine
NAT10: N-acetyltransferase 10
H3K18: Histone H3 lysine 18
H4K12: Histone H4 lysine 12
METTL3: Methyltransferase-like 3
H3K18la: Histone H3 lysine 18 lactylation
GCLC: Glutamate-Cysteine Ligase Catalytic Subunit
CAFs: Cancer-Associated Fibroblasts
FTH1: Ferritin Heavy Chain 1
SUMO2: Small Ubiquitin-like Modifier 2
PCBP2: Poly(rC)-binding protein 2
HDAC1: Histone Deacetylase 1
TIME: Tumor Immune Microenvironment
ICD: Immunogenic Cell Death
APCs: Antigen-Presenting Cells
HMGB1: High Mobility Group Box 1
CRT: Calreticulin
MHC II: Major Histocompatibility Complex Class II
4-HNE: 4-Hydroxynonenal
NF-κB: Nuclear factor kappa-light-chain-enhancer of activated B cells
IL-12: Interleukin 12
PGE₂: Prostaglandin E₂
TAMs: Tumor-Associated Macrophages
JAK: Janus kinase
STAT1: Signal transducer and activator of transcription 1
TME: Tumor Microenvironment
HSPs: Heat Shock Proteins
MDSCs: Myeloid-Derived Suppressor Cells
PD-L1: Programmed Death-Ligand 1
Drp1: Dynamin-Related Protein 1
iNOS: inducible Nitric Oxide Synthase
NO•: Nitric oxide radical
STAT3: Signal Transducer and Activator of Transcription 3
PMN-MDSCs: Polymorphonuclear Myeloid-Derived Suppressor Cells
KEAP1: Kelch-Like ECH-Associated Protein 1
ASAH2: N-acylsphingosine amidohydrolase 2
NK: Natural Killer
PD-1: Programmed cell death protein 1
Tregs: Regulatory T cells
TCR: T-cell receptor
PI3K: Phosphatidylinositol 3-kinase
CPT1A: Carnitine Palmitoyltransferase 1A
CTLA-4: Cytotoxic T-lymphocyte-associated protein 4
SAPE-OOH: 1-Stearoyl-2-arachidonoyl-sn-glycero-3-phosphoethanolamine hydroperoxide
mTORC2: Mammalian Target of Rapamycin Complex 2
NKG2D: NK group 2D
RORC: RAR-related orphan receptor C
O-GlcNAcylation: O-linked β-N-acetylglucosamine modification
FOXK1: forkhead box K1
PROTAC: Proteolysis-Targeting Chimera
PSAT1: Phosphoserine Aminotransferase 1
TILs: Tumor-infiltrating lymphocytes
NQO1: NAD(P)H quinone dehydrogenase 1
